# Supplementary material for: Full-length genomes evolve in parallel in a long-term evolution experiment with phage φX174
Source: PLoS Biol. 2026 Oct 29;24(10):e3004064. doi: 10.1371/journal.pbio.3004064 (PMC13743035; doi:10.1371/journal.pbio.3004064)
Supplement: Supplementary file 1 [file pbio.3004064.s001.pdf]

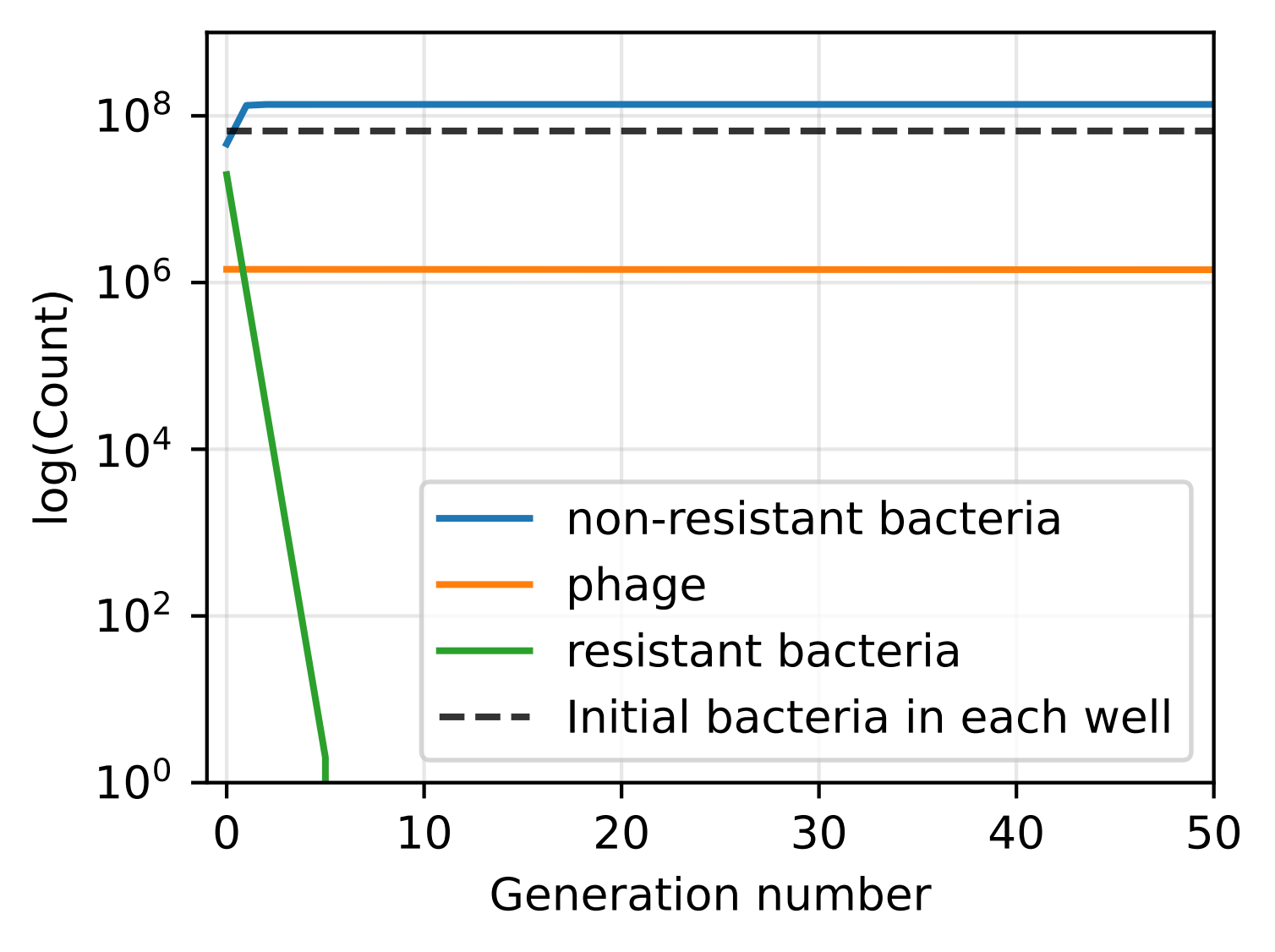

log(Count)
10^8
10^6
10^4
10^2
10^0
0
10
20
30
40
50
Generation number
non-resistant bacteria
phage
resistant bacteria
Initial bacteria in each well
