# Supplementary material for: Full-length genomes evolve in parallel in a long-term evolution experiment with phage φX174
Source: PLoS Biol. 2026 Oct 29;24(10):e3004064. doi: 10.1371/journal.pbio.3004064 (PMC13743035; doi:10.1371/journal.pbio.3004064)
Supplement: Supplementary file 2 [file pbio.3004064.s003.pdf]

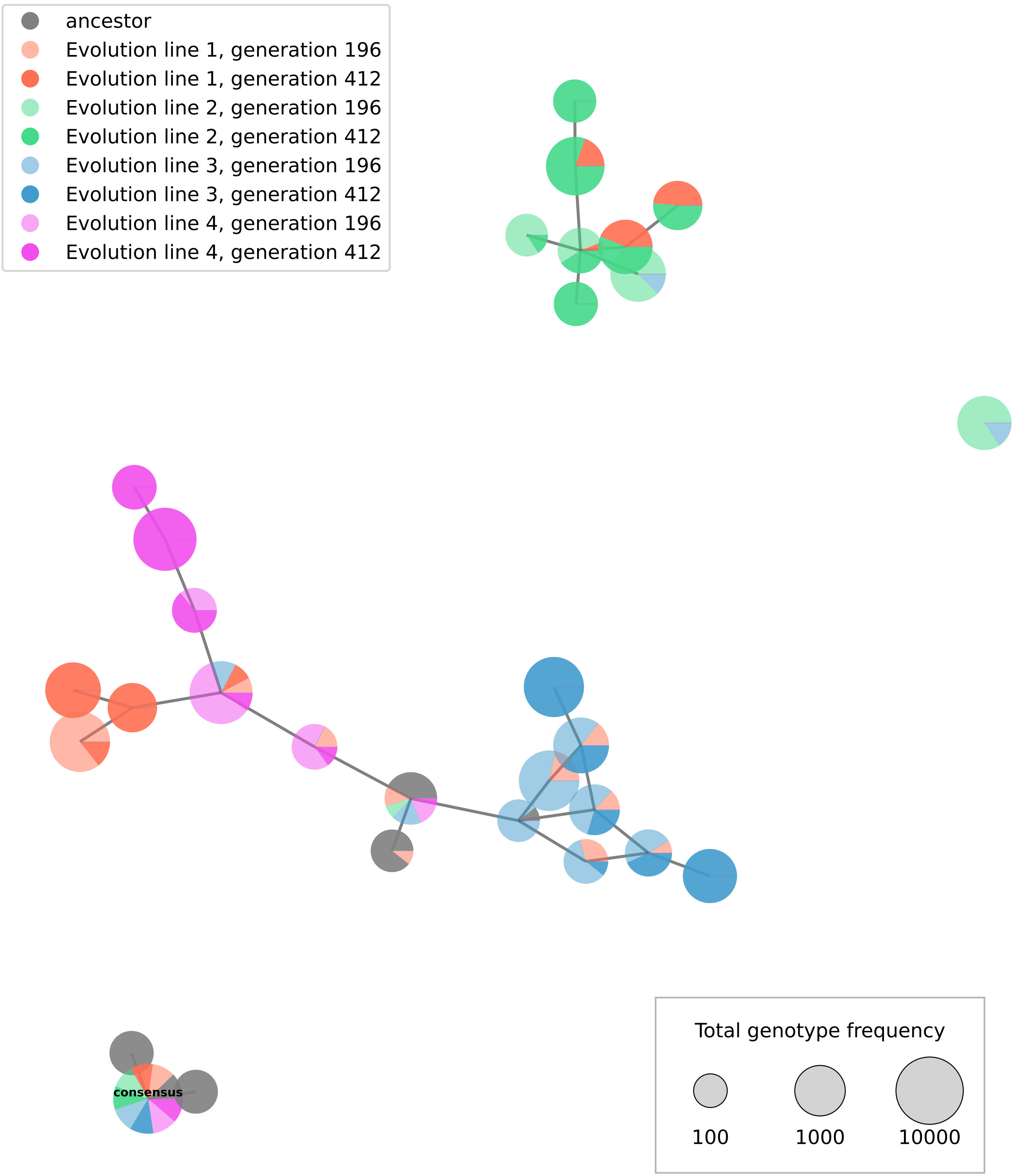
ancestor
Evolution line 1, generation 196
Evolution line 1, generation 412
Evolution line 2, generation 196
Evolution line 2, generation 412
Evolution line 3, generation 196
Evolution line 3, generation 412
Evolution line 4, generation 196
Evolution line 4, generation 412
consensus
Total genotype frequency
100
1000
10000
