# Supplementary material for: Full-length genomes evolve in parallel in a long-term evolution experiment with phage φX174
Source: PLoS Biol. 2026 Oct 29;24(10):e3004064. doi: 10.1371/journal.pbio.3004064 (PMC13743035; doi:10.1371/journal.pbio.3004064)
Supplement: Supplementary file 3 [file pbio.3004064.s004.pdf]

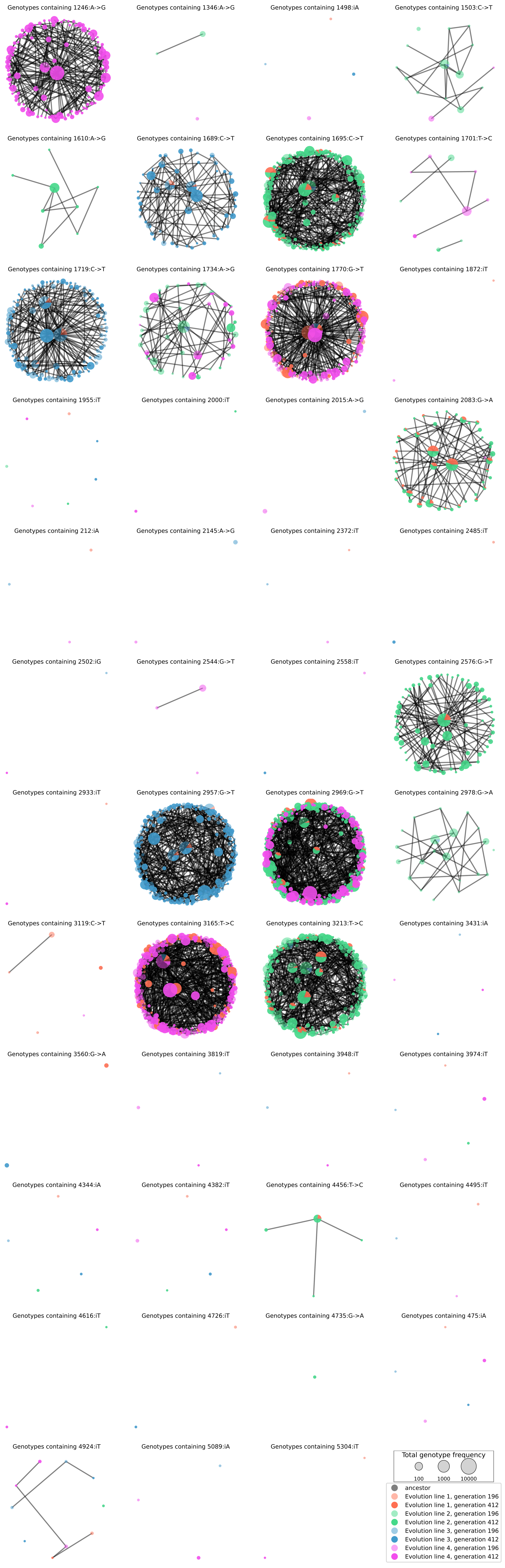
Genotypes containing 1246:A->G
Genotypes containing 1346:A->G
Genotypes containing 1498:iA
Genotypes containing 1503:C->T
Genotypes containing 1610:A->G
Genotypes containing 1689:C->T
Genotypes containing 1695:C->T
Genotypes containing 1701:T->C
Genotypes containing 1719:C->T
Genotypes containing 1734:A->G
Genotypes containing 1770:G->T
Genotypes containing 1872:iT
Genotypes containing 1955:iT
Genotypes containing 2000:iT
Genotypes containing 2015:A->G
Genotypes containing 2083:G->A
Genotypes containing 212:iA
Genotypes containing 2145:A->G
Genotypes containing 2372:iT
Genotypes containing 2485:iT
Genotypes containing 2502:iG
Genotypes containing 2544:G->T
Genotypes containing 2558:iT
Genotypes containing 2576:G->T
Genotypes containing 2933:iT
Genotypes containing 2957:G->T
Genotypes containing 2969:G->T
Genotypes containing 2978:G->A
Genotypes containing 3119:C->T
Genotypes containing 3165:T->C
Genotypes containing 3213:T->C
Genotypes containing 3431:iA
Genotypes containing 3560:G->A
Genotypes containing 3819:iT
Genotypes containing 3948:iT
Genotypes containing 3974:iT
Genotypes containing 4344:iA
Genotypes containing 4382:iT
Genotypes containing 4456:T->C
Genotypes containing 4495:iT
Genotypes containing 4616:iT
Genotypes containing 4726:iT
Genotypes containing 4735:G->A
Genotypes containing 475:iA
Genotypes containing 4924:iT
Genotypes containing 5089:iA
Genotypes containing 5304:iT
Total genotype frequency
100
1000
10000
ancestor
Evolution line 1, generation 196
Evolution line 1, generation 412
Evolution line 2, generation 196
Evolution line 2, generation 412
Evolution line 3, generation 196
Evolution line 3, generation 412
Evolution line 4, generation 196
Evolution line 4, generation 412
