# Supplementary material for: Full-length genomes evolve in parallel in a long-term evolution experiment with phage φX174
Source: PLoS Biol. 2026 Oct 29;24(10):e3004064. doi: 10.1371/journal.pbio.3004064 (PMC13743035; doi:10.1371/journal.pbio.3004064)
Supplement: Supplementary file 4 [file pbio.3004064.s007.pdf]

	A	EL1	EL2	EL3	EL4
A	0.00000	0.00021	0.00020	0.00022	0.00019
EL1	0.00153	0.00000	0.00182	0.00095	0.00098
EL2	0.00138	0.00168	0.00000	0.00065	0.00059
EL3	0.00132	0.00077	0.00056	0.00000	0.00062
EL4	0.00145	0.00104	0.00069	0.00084	0.00000
