# Supplementary material for: Full-length genomes evolve in parallel in a long-term evolution experiment with phage φX174
Source: PLoS Biol. 2026 Oct 29;24(10):e3004064. doi: 10.1371/journal.pbio.3004064 (PMC13743035; doi:10.1371/journal.pbio.3004064)
Supplement: Supplementary file 6 [file pbio.3004064.s010.pdf]

Point-by-point Responses to Reviewers

We would like to thank the reviewers for their thoughtful points of criticism. We have addressed them point-by-point (see below).

There was only one remaining main point raised by Reviewer #2 who is concerned that it might have been contamination rather than true parallel evolution that gave rise to our findings. They suggested to investigate the genetic background of the parallel mutations and combinations of mutations. We are grateful for this suggestion as it gave us the opportunity to put these concerns to rest. In brief, we found that the parallel mutations are mostly on different genotypes in each evolution lines, strongly supporting parallel evolution (see below for more details).

Below, the points of the reviewers' are quoted in italics.

Reviewer #1:

[identifies himself as David Rasmussen]

> *The authors have addressed my previous concerns and performed additional analyses (e.g. masking sites undergoing convergent evolution) that provide compelling evidence that parallel mutations are driving phylogenetic errors.*
>
> *Minor comments:*
>
> *In the Introduction it might be good to cite relevant inference frameworks when introducing the field of phylodynamics.*

Response: Thank you very much for that suggestion. We now include a sentence in line 13 with a reference to a recent review paper that discusses relevant phylodynamic inference schemes and software packages such as BEAST 1/2/X, and treetime (which we use), and others. The sentence reads (lines 13-15):

"*Several phylodynamic inference frameworks and implementations exist that differ in their assumptions about the biological processes and the statistical approach to fitting the phylodynamic models (reviewed in Featherstone et al 2022).*"

> *Throughout: "phylodynamics inference" should probably be "phylodynamic inference".*

Response: Thank you very much for spotting this. It has been corrected throughout the manuscript.

> *Discussion line 242: "Thus, the distributions of migration rate estimates in Fig 6 can also be interpreted as the distribution of the inaccuracy of the reconstructed phylogenetic trees." -- I agree that the distribution of migration rates in some way quantifies the inaccuracy of phylogenetic reconstruction. But maybe it would be more clear to link the number of imputed migration rates to the number of discordant edges*

(i.e. subtree-prune-regraft moves) between the true and reconstructed trees? I think this would be more precise way to quantify inaccuracy since every edge imputed to have a migration event must result from a reconstruction error.

Response: While we agree with the reviewer that this would be an interesting analysis, the central problem we would face is that we do not have the “true tree” as our study is experimental rather than computational. The closest we could get to the true evolutionary history would be a reconstruction of the sequences evolution line by evolution line and then concatenate them to a larger tree with four main branches. This would still be associated with error within each branch.

Moreover, for a good comparison with the migration estimates, we would probably need to reconstruct these approximated true trees for each subsample of the data that we drew for each treetime migration rate estimate.

Due to the inherent uncertainties associated with this analysis and the substantial computational challenges we foresee, we decided not to perform this analysis. If the reviewer insists, we would be happy to implement this, but then would need more time.

Discussion lines 250-252: "it will be extremely challenging to disentangle whether mutations co-occur in multiple locations due to parallel". -- It may be difficult but probably not impossible. If most polymorphic sites are not undergoing convergent evolution, one could reconstruct a first-pass tree from the full alignment and then use that initial tree to identify sites with a high degree of homoplasy i.e. convergent evolution. One could then filter or mask these sites from the alignment to get an improved phylogenetic reconstruction. Similar approaches are already used to identify and mask recombination events.

Response: We thank the reviewer for challenging our discussion of how feasible it is to correct the bias arising by parallel evolution by masking sites with homoplasies. In our view, the procedure the reviewer outlines could work in systems in which we have only parallel evolution but no migrations (as our experimental system). But if migration also occurs, or is at least a possibility, the procedure has the risk of deleting the strongest signals for migration. We have, nevertheless, revised our discussion of this issue and now present a correction of these biases as challenging rather than not feasible (lines 267-275):

“Although this analysis clearly shows that parallel mutations are the cause of the bias, turning this insight into a correction of the biases comes with challenges. First, in most cases the sites that can harbor adaptive mutations are not known. In our experiments, for example, we did not impose any selection intentionally, and most of the parallel mutations were unanticipated and unknown. Exceptions are known antiviral drug-resistance mutations that are commonly masked when reconstructing the transmission history of HIV-1. Second, in epidemiological systems with potential migration between locations, the challenge is to disentangle whether mutations co-occur in multiple locations due to parallel evolution or migration. Masking parallel mutations comes at the risk of disregarding footprint of true migration.”

Original text for reference:

“Although this analysis clearly shows that parallel mutations are the cause of the bias, it is not a viable solution to the problem because parallel mutations are difficult to identify. In our experiments we did not impose any selection intentionally, and most of the parallel mutations were unanticipated and unknown. In epidemiological systems with potential migration between locations it will be extremely challenging to disentangle whether mutations co-occur in multiple locations due to parallel evolution or migration. Exceptions are known antiviral drug-resistance mutations that are commonly masked when reconstructing the transmission history of HIV-1.”

> *Discussion lines 285-287" "however, recombination is unlikely to significantly distort the inferred evolutionary 285 histories because the genotypes in our experiment are highly related, and correct phylogenies can be successfully inferred in such a regime" -- I'm not sure I agree. Phylogenetic inference is generally the most challenging when genetic diversity is low such that recombination becomes difficult to detect and therefore impossible to account for.*

Response: We thank the reviewer for this point. We realized we had been vague about the reasons why recombination will not significantly distort our study results. While we agree that recombination will be difficult to detect and correct in this regime, this aspect is not central as we are not attempting to correct for recombination in our study. More important are the effects of recombination on viral diversification and parallel evolution. We strongly believe that recombination – because of the limited diversity of our viral genotypes - will introduce mostly single mutations not unlike point mutation, and hence have a similar diversifying effect. (In high diversity scenarios, recombination can introduce dozens of mutations at once, leading to a severe amplification of diversification.) Even more importantly, recombination acts within each evolution lines and will therefore not contribute to parallel evolution. We therefore rephrased this sentence to (lines 306-308):

“In our experimental setting, however, recombination is unlikely to significantly distort the inferred evolutionary histories because it would act only within each evolution line and hence not contribute to parallel evolution.”

Reviewer #2:

> *First, the authors have done a great job of addressing many of my concerns.*

Response: We are very glad to hear that!

> *My main remaining concern is about cross-contamination and parallel evolution. I acknowledge the experimental controls described in the manuscript (page 73), and I agree that they make contamination less likely. However, they do not rule it out. The manuscript would be considerably strengthened by explicitly demonstrating that the repeated multi-mutation genotypes represent independent evolutionary events rather than shared ancestry due to contamination. The authors have a simple way to test this using their existing data, so why not do it?*
>
> *The key point is that cross-contamination and parallel evolution make fundamentally different predictions at the whole-genome level. Cross-contamination transfers*

complete genomes. If an evolved virus from one evolution line is accidentally introduced into another, it carries with it its entire evolutionary history: not only the focal adaptive mutations, but also every other mutation that accumulated in that lineage, including neutral and hitchhiking variants. In contrast, genuine parallel evolution is expected to produce repeated adaptive mutations on independent genetic backgrounds. Selection may repeatedly favour mutations in the same genes, or even the same combinations of mutations, but the remainder of the genome should differ because each evolution line has accumulated its own independent set of mutations.

Because the authors have whole-genome haplotype information, they are uniquely positioned to distinguish between these alternatives. For each repeated multi-mutation genotype, they should simply compare the complete mutational profiles across the independent evolution lines. If the genomes observed in different evolution lines are genetically indistinguishable across the complete genome, and have multiple differences to the ancestral genome, this would be difficult to reconcile with independent evolution and would instead be more parsimoniously explained by cross-contamination or sample mix-up. Conversely, if the shared adaptive mutations occur within otherwise distinct genomic backgrounds, this would provide compelling direct evidence that the mutations arose independently and would substantially strengthen the claim of parallel evolution.

My concern is that the manuscript makes the unusually strong claim of repeated evolution of whole-genome genotypes, yet the one analysis that would most directly distinguish independent parallel evolution from contamination is not presented. Instead, the manuscript relies on indirect arguments against contamination (experimental controls, spatial separation of evolution lines, genotype frequencies, etc.). These observations do not answer the central genomic question that the authors are uniquely positioned to address with their existing whole-genome data.

This strikes me as a straightforward analysis that can be performed using the existing sequencing data. Given the strength of the claim, I think presenting this analysis explicitly would greatly strengthen the conclusions and remove any lingering ambiguity regarding contamination. At present, this critical distinction is not evident from either the manuscript or the response to reviewers.

Response: We very much appreciate that the reviewer is persisting on this central issue of our study and are very grateful for the outline of an analysis that sheds light on whether the mutations are truly parallel.

We have conducted the analysis the reviewer suggests: we looked at each of the 47 mutations that appear in multiple evolution lines and determined the genotypes in which they are found. (Unlike in the Lenski experiments with bacteria, our evolution lines harbor hundreds of concurrent genotypes at generation 196 and 412, and probably throughout the entire experiment.) We then determined what fraction of these genotypes are shared across the lines. A high fraction of shared genotypes would support cross-contamination, while a low fraction supports parallel evolution.

We find that the fraction of shared genotypes of mutations that appear in multiple lines is 2.2%. If we weight the average by the frequency of each distinct genotypes it is 3.5%. Thus,

the vast majority of genotypes in which shared mutations occur are unique to the evolution line, indicating that they have mostly not evolved on the same genetic background, and supporting true parallel evolution over cross-contamination.

We performed this analysis of the genetic background for combinations of 2, 3, and 4 parallel mutations also. Similar as for single parallel mutations, only a minority of the genotypes that harbored the combination were found in multiple lines. The fractions are 6.1%, 18%, and 27%, for combinations of 2, 3 or 4 mutations respectively. If we weight the average by the number of genotypes, the fractions are 6.5%, 13%, and 20%, for combinations of 2, 3 or 4 mutations respectively.

We now present this analysis in the supplementary file “stats-for-paper-rrr.html”, along with tables detailing the number of genotypes (total and shared across multiple evolution lines) that harbor each of the parallel mutations or combinations of mutations.

To visualize the genetic distances between these genotypes, we have complemented this analysis by genotype network graphs for each of the 47 mutations. This is now S4 Fig and pasted below for convenience.

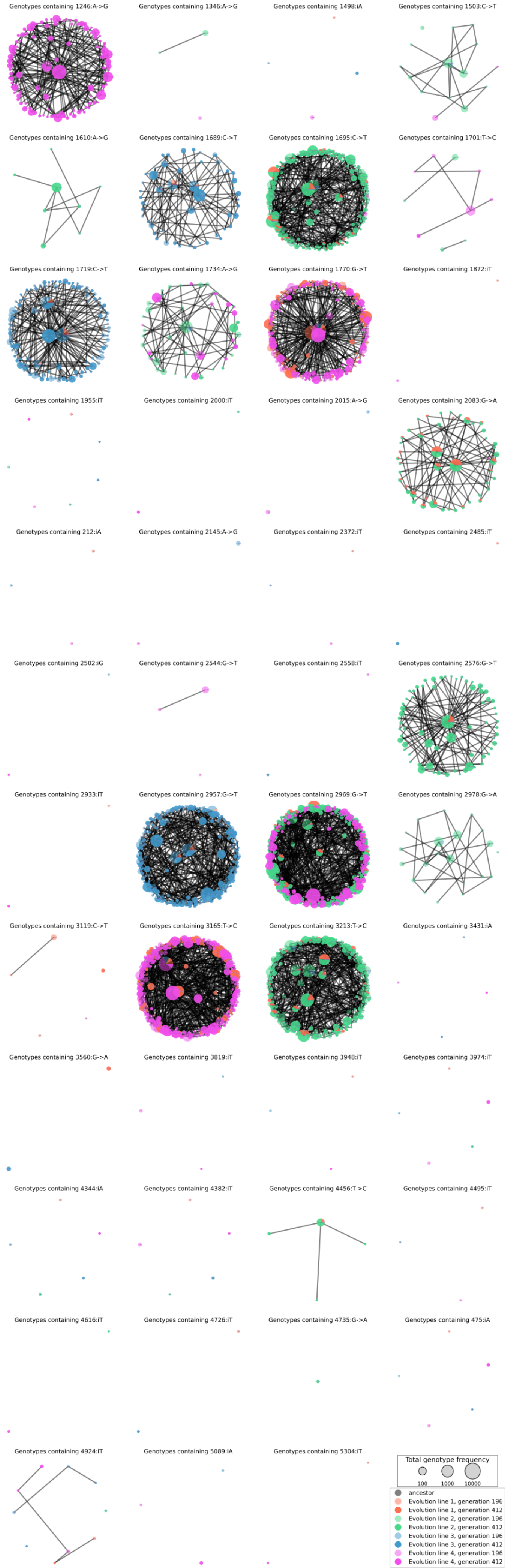

S4 Fig: Genotype network plots of genotypes containing specific parallel mutations. For each parallel mutation (those that occur in at least two different lines), a genotype network plot is shown for all genotypes present during the experiment containing that parallel mutation. The networks are presented in the same way as in S3 Fig. There is a large variance in the number of genotypes containing a specific parallel mutation, though in general genotypes are found mostly in a single evolutionary line. This is evidence against contamination, and supports that these mutations are the result of true parallel evolution.

In the revised manuscript, we are summarizing this analysis in the main text as a further argument that the mutations that are found in multiple lines have arisen by parallel evolution (in lines 194-208) where we now write:

“As for the parallel genotypes, the parallel mutations and mutation combinations are very unlikely to have arisen by cross-contamination. The most compelling piece of evidence against contamination and in favor of true parallel evolution is that the parallel mutations are found on mostly different genomic backgrounds in different evolution lines. First we investigated how diverse the genotypes are that harbor the 47 single parallel mutations in each evolution line. We determined that each mutation is found on multiple genotypes but that the majority of these genotypes differ across evolution lines. On average only 2.5% of the genotypes are the same across the evolution lines in which a parallel mutation is detected. If we weight the average by the frequency of each distinct genotype, the fraction is 3.5% - similarly low. To visualize the low overlap of these genotypes across evolution lines, we have complemented this analysis by genotype network graphs for each of the 47 mutations (S4 Fig). We also performed this analysis of the genetic background for combinations of 2, 3, and 4 parallel mutations. Similar as for single parallel mutations, only a minority of the genotypes that harbored the combination were found in multiple lines. The fractions are 6.1%, 18%, and 27%, for combinations of 2, 3 or 4 mutations respectively. If we weight the average by the number of genotypes, the fractions are 6.5%, 13%, and 20%, for combinations of 2, 3 or 4 mutations respectively.”

We also added the network graph described above as a new supplementary Figure S4.

> *Minor points:*
>
> *In the abstract, why not simply state that you follow the genetic diversification of four bacteriophage populations for 412 generations? You cannot rule out that selection is acting on the population. At present, the authors emphasize that they are not actively imposing selection, while simultaneously acknowledging that selection is probably operating through the bacterial cell environment. Moreover, the principal conclusion of the study is that selection is indeed shaping the evolutionary trajectories, so this repeated qualification seems unnecessary.*

Response: The main reason we emphasize that we did not actively impose selection is that neutrality is a core assumption of phylogenetic reconstruction and phylodynamics – an inference scheme we set out to test. Not imposing strong selection is also not the norm in experimental evolution studies that rarely try to operate in a neutral regime.
So we did not simply “follow the genetic diversification”. We had an over-arching aim of assessing phylodynamic methods. (In this aspect, our study differs from the long-term evolution experiment by Lenski et al.)

Our reading of the reviewer’s point here is that they do not believe we state something wrong. They simply think that there would be a more concise way to describe our study. Instead of following the reviewer’s advice, we are now emphasizing in the revised Abstract and in the Introduction that a neutral regime is most consistent with the assumptions of phylodynamic inference – which we set out to test.

Specifically we now write In the Abstract:

“One of our goals was to assess phylodynamic methods that use genetic information to infer key parameters of the population history. Because phylodynamic methods assume neutral evolution, we did not actively impose any selection pressures in our evolution experiment (in addition to those inherent in the bacterial cell environment).”

An in the Introduction we added (lines 17-21):

“In particular, evolution is mostly assumed to be neutral, and selection pressures and their influence on reconstructing genealogies are not taken into consideration. Most importantly, phylodynamic inference methods have not yet been experimentally assessed. To this end, we need to generate data that allow us to trace the evolutionary dynamics of viruses in fine detail in settings in which we do not impose strong selections pressures.”

> *I agree with the authors that Figure 4 presents an exceptionally rich and novel dataset, however, in my veiw, I do not think it is appropriate to state that evolution is observed in "unprecedented detail," as this is an unverifiable claim.*

Response: While we do not know of any study that reported viral evolution in this detail, we agree that the statement that we observed viral evolution in “unprecedented detail” is technically unverifiable, and changed the wording to “extraordinary detail” in the Abstract and at the end of the Introduction.

> *Line 202: typo — "ancenstor".*

Response: Corrected!
