# Supplementary material for: Full-length genomes evolve in parallel in a long-term evolution experiment with phage φX174
Source: PLoS Biol. 2026 Oct 29;24(10):e3004064. doi: 10.1371/journal.pbio.3004064 (PMC13743035; doi:10.1371/journal.pbio.3004064)
Supplement: Supplementary file 8 [file pbio.3004064.s008.docx]

# Point-by-point Responses to Reviewers

We would like to thank the reviewers for their thought-provoking criticism. We have addressed them point-by-point (see below). While we disagree with the main point of criticism that bacteria could have evolved resistance to phage in our experiment, we believe that the reviewers’ input made our paper stronger.

As we stated in our appeal, we disagree with the main criticism of the Reviewers #2 and #3 that our study is “flawed” because we were very likely selecting for resistant bacteria in the evolution experiment. In the revised submission, we present computer simulations — based on straight-forward principles of microbial population biology — that show that selecting for resistant bacteria is very unlikely in our setting. This is due to the fact that each passage is only 26 minutes long, and the new well contains an overwhelming number of wildtype bacteria from the stock that has not seen phages. We have included plots of the output of the simulation that shows that the level of resistant bacteria will not increase appreciably, and will actually be washed out in most conceivable scenarios (see new Figure S1 in the revised paper and the responses to point 1 of Reviewer #3).

Below, the points of the reviewers’ are quoted in italics.

## Reviewer #1:

*[identifies himself as David A. Rasmussen]*

*Bons el. combine experimental evolution of the phage phiX1743 with long-read (whole genome) sequencing. These methods allow the authors to track the evolutionary dynamics of full-length haplotypes in the viral population, which few viral deep sequencing studies have done before. One key result is that there is a high degree of convergent evolution across multiple sites in the genome. While such convergent evolution in phage evolution studies has been reported before (Bull et al., 1997), its particularly surprising here as the phage populations were not subjected to any novel selection pressure, host or environment. The extent of convergent evolution has major implications for reconstructing viral phylogenies accurately, and therefore downstream inferences from drawn from these phylogenies.*

*Overall, the work reported here is of high quality and I only have a few minor comments where certain arguments could be better supported by additional analysis. While I don't want to dictate how the authors present their work, I also thought that the emphasis on migration rates biases somewhat misses the larger story here about how convergent evolution could bias phylogenetic inference more generally.*

*Major comments:*

*1) Accuracy of phylogenetic reconstructions: Presumably migration rates are estimated to be non-zero in the phylodynamic analyses because samples from different evolutionary lines are interspersed with one another in the reconstructed trees. This suggests there is a bigger underlying problem with phylogenetic inference in the presence of convergent evolution, consistent with the problem of "exceptional convergent evolution" identified by Bull et al. (1997). This to me is the bigger story here since nearly all downstream phylodynamic and demographic inference methods assume that the trees can be reconstructed reasonably accuracy. It might therefore be interesting to quantify the degree of phylogenetic error caused by convergent evolution. This seems fairly straightforward in this case as you know there's a reconstruction error any time samples from the same evolutionary lineage fall outside the same clade, such that the extent of polyphyletic relationships among samples from the same line can be used to quantify the extent of phylogenetic error.*

**Response**: We agree with the reviewer that the inaccuracy of the phylogenetic reconstruction is at the heart of the bias in phylodynamic analysis that we identify. We strongly believe it is important to document how the parallel evolution trickles through the phylodynamics analysis pipeline, and how large the resulting error is. The bias we identify is quite substantial (amounting to a 68% probability of every genome to migrate during the experiment), especially since we did not impose any selection intentionally and, as a result, parallel evolution was not as pronounced as it could have been.

We really like the reviewers suggestion to quantify the accuracy of the phylogenetic reconstruction by counting the "swaps" of evolutionary lines along each branch of the tree. On careful consideration, we realized that the migration rate estimate is basically that: the number of swaps divided by the tree height. We added a paragraph to that effect in the Discussion (lines 321-326).

**Revised text**:

"The inaccuracy of the reconstructed phylogenetic tree could be quantified by the number of times a lineage assignment switches from one evolution line to another. This quantity, however, is not very different from the migration rate estimate, which is, in a nutshell, derived from the number of such switches divided by the tree height (Lemey et al PLOS Comp Biol 2009). Thus, the distributions of migration rate estimates in Figure 6 can also be interpreted as the distribution of the inaccuracy of the reconstructed phylogenetic trees.”

*2) Source of phylogenetic error: It has become standard practice in some fields to mask or remove sites undergoing convergent evolution from alignments prior to phylogenetic analysis i.e. sites conferring drug resistance mutations in viral genomes. In order to more definitively show that convergent evolution is the source of phylogenetic error and biases in migration rates, it might therefore be interesting to mask the sites undergoing convergent evolution in the alignment. Showing that masking these sites eliminates the upwards bias in migration rates would provide strong support that it is actually parallel evolution driving this bias.*

**Response**: We thank the reviewer for this suggestion! We have conducted a phylodynamic analysis masking all mutations that appeared in multiple lines, i.e. parallel mutations, or just those that appeared in 3 or 4 lines which we call "excess parallel mutations" because only these are more numerous than neutral simulations suggest (see Figure 5A).

We find that the significant bias in the migration rate estimate disappears in both cases, clearly showing that parallel mutations are the source of this bias.

In the revised manuscript, we describe these additional analyses (lines 294-301) and extended Figure 6 by the distributions of migration rate estimates based on our sequencing data with masked (excess) parallel mutations. For convenience, we are showing the revised Figure 6 and its legend here:

We would like to note that masking parallel mutations is not a viable solution to the bias problem. It will work for only a small subset of parallel mutations, such as known drug resistance mutations in HIV-1 that the reviewer mentions. In our experiments we did not impose any selection intentionally, and most of the parallel mutations were unanticipated and unknown. In epidemiological systems with potential migration between locations it will be extremely challenging to disentangle whether mutations co-occur in multiple locations due to parallel evolution or migration.

In the revised manuscript, we introduced a paragraph in the Discussion that elaborates on this issue (lines 327-338):

**Revised text**:

"As direct evidence that parallel evolution leads to the biases in phylodynamic inference, we conducted analyses with datasets that either did not include the 26 genotypes that evolved in multiple lines, or masked (excess) parallel mutations. While the analysis without the 26 parallel genotypes did not correct the bias, masking parallel mutations did (see Figure 6). Although this analysis clearly shows that parallel mutations are the cause of the bias, it is not a viable solution to the problem because parallel mutations are difficult to identify. In our experiments we did not impose any selection intentionally, and most of the parallel mutations were unanticipated and unknown. In epidemiological systems with potential migration between locations it will be extremely challenging to disentangle whether mutations co-occur in multiple locations due to parallel evolution or migration. Exceptions are known antiviral drug-resistance mutations that are commonly masked when reconstructing the transmission history of HIV-1.”

*3) Fitness of genotypes: The authors argue that the mutations that repeatedly reoccur in the same haplotype are likely positively epistatic. This claim seems like it could be better supported. One could estimate the relative fitness (selection coefficients) acting on both single and double mutants (e.g. by using genetic time series methods) to show that the selective advantage of the double mutants is more than the additive effects of the single mutants.*

**Response**: We thank the reviewer for supporting the idea of estimating fitness and epistatic effects using sequence data over time. We are aware of these methods and have been writing grant proposals to generate the data allowing to apply these methods. We have been thinking of MPL (Sohail et al *Nat Biotech* 2020, *Mol Biol Evol* 2022) specifically and are in contact with the Barton group about this.

The reason we need to generate new data (and more funding) for this is that the sequencing data we present in this manuscript have been obtained at only two time points during the long-term evolution experiment that are 200 generations apart. For methods estimating fitness and epistatic effects from their frequencies, one requires more frequent sampling. Essentially, one needs to catch mutations at frequencies between 0 and 1 multiple times for such an estimation to work.

For the time being, the indirect evidence we provide about epistatic effects is all we can offer. But we mention the time series approach and its requirements in the Results (lines 245-248):

**Revised text**:

"More direct evidence for positive epistasis between mutations could be gained from more fine-grained data on the frequency of genotypes over time. This, however, will require to sample and sequence more frequently than every 200 generations.”

*Minor comments:*

*Line 44-45: "phylodynamic inference methods have not yet been experimentally assessed." -- One could argue that they have been extensively experimentally assessed by simulation, just not with carefully controlled in vivo experiments.*

**Response**: We thank the reviewer for this point. We certainly do not intend to inflate the lack of testing of phylodynamic methods. We would not, however, call simulations "experimental" though.

In sentences preceding the quote we state clearly that phylodynamic methods have been assessed by simulation and motivate experimental testing (now lines 43-46): “These inference schemes are usually tested on data that were simulated according to the assumption of the inference models. In particular, when testing these schemes, selection pressures and their influence on reconstructing genealogies are not taken into consideration.”

Additionally, in most cases phylodynamic methods are tested on simulated data that conform to the model assumptions, especially the assumption of neutral evolution. We know of only the PANGEA study (Ratmann et al Mol Biol Evol 2017) that tested phylodynamic methods on so-called "mis-specified" simulation models, i.e. simulation models that did not conform to the model used for inference. (In Ratmann et al the simulation models were "mis-specified" by including demographic factors such as age and household structure, and transmission differences between various demographic groups.). However, sometimes phylodynamic methods are even tested directly on simulated trees, rather than on simulated genetic sequence data, thus neglecting the inaccuracies of phylogenetic reconstruction that can occur when applying the methods to epidemiological data (e.g. Lemey et al 2009 PLOS Comp Biol).

We feel that we stated all this clearly in a dedicated paragraph in the Discussion (now lines 339-352).

As an aside, we use *in vivo* exclusively for the dynamics of viruses within a living host. We would describe our experimental system as either *in vitro*, or as *in cell culture*. But conventions could differ.

*Line 52-53: "high-throughput sequencing can detect low frequency mutations, but at the cost of loosing linkage information" -- I don't think this is inherent to HTS, but specific short-read sequencing technologies. Long read platforms would not suffer from the same constraints on assembling haplotypes.*

**Response**: This is a valid point. We rephrased that sentence (now lines 54-56).

**Revised Text:**

“On the other hand, early high-throughput sequencing technology could detect low frequency mutations, but generated short reads and thus did not provide linkage information ...”

*This might just be an issue of semantics, but it sounds odd to claim these populations are evolving without selection pressures since even in a stable environment any phage population would be under very strong selection to survive, replicate, compete for hosts, and so on. Maybe it would be more clear to say that these populations were evolved in the absence of "novel selection pressures"?*

**Response**: We certainly did not mean to suggest there are no selection pressures in our experimental system (which we, in part, document by analyzing parallel evolution patterns). We were aiming to create a system as close to neutral as possible to be in-line with the assumptions of phylodynamics. For that reason, we did not intentionally exert selection pressures by exposing the phages to a novel environment (as the reviewer points out), or by antibodies or similar. That is what we meant in the Abstract when we state that we evolved the phages "without imposing any direct selection pressure".

We rephrased this to "without actively imposing any selection pressures (in addition to those inherent in the bacterial cell environment)" (now lines 12-13), and hope this phrasing is less misleading.

## Reviewer #2:

*In this study, the authors performed an interesting evolution experiment using bacteriophage and bacterial hosts in a highly novel design. They transferred phage populations onto naive bacteria every 26 minutes across 412 sequential transfers, using a robotic liquid handler. They complemented this experiment with extensive high-quality DNA sequencing and simulations to report on the evolutionary dynamics of evolution in replicate phage populations. This approach represents a new and interesting direction for experimental evolution and has generated a valuable dataset. While the work is innovative and the data are impressive, I have questions primarily about the analysis and the strength of some of the conclusions.*

*Comments:*

*Line 34 Variant should be variants.*

*L49 "On the one hand"*

*L60 sentence requires a period.*

**Response:** We thank the reviewer for identifying these typos. They have been corrected.

*L84 perhaps "the rationale for not intentionally applying selection" would be clearer?*

**Response:** We changed this phrase to what the reviewer suggested. (See also the last point of Reviewer #1 above.)

*L102 The authors write that "We obtain over 110'000 reads covering the entire genome of φX174, representing" over 31'000 unique genotypes" Is this evenly distributed across the 8 populations you sequenced? (4 replicates x 2 time points)? I think the authors should report the coverage per replicate population and time point. If the coverage per replicate population is approximately equal, then you are getting at least 10,000 reads per population. Since your maximum read length is 5kb (the length of the phage genome), this puts you at around 10000x coverage for the population. What is your Qscore for each of your genomes? If it is Q30, we expect 5 errors for each 50kb genome - I believe Qscore for PacBio is incorporates the benefits of the smrt cell repeated rolling circle sequencing approach. This means many errors will be included in your analysis. Why not combine reads from multiple smrt cells to so that you can get the benefits of high coverage?*

**Response:** We thank the reviewer for raising these points.

The yield of full-genome reads was comparable in each sample, ranging from 8866 to 16646 reads. Specifically, we obtained 9169 full-genome reads in the ancestor sample, 9888, 11125, 16522, and 14001, full-genome reads from the samples at generation 196 for evolution line 1, 2, 3, and 4 at generation 196, respectively, and 8866, 13088, 13429, and 16646 full-genome reads from the samples from evolution line 1, 2, 3, and 4 at generation 412, respectively.

This information can be found in the file “R-code/code/stats-for-paper-rrr.html” included in the supplementary zip-archive “data+code-for-reviewers-May25-2026.zip”. The file is generated from a Rmarkdown worksheet “R-code/code/stats-for-paper-rrr.Rmd” that is designed to allow interested readers (and reviewers) to adapt it and extract any such statistics from our rich dataset. We intentionally provide access to our data in this interactive format because we anticipated that we will not be able to list all conceivable statistics in the main paper.

The quality scores were stated in the Materials and Methods. For the analysis, we considered only circular consensus sequences with a quality score higher than 47. This translates to an error rate of 2 × 10^−5^ per nucleotide, or to less than one mis-called nucleotide per genome. The reason for still having over 110’000 sequences with this high score is the long polymerase read length of PacBio (>38kb in our experiment) and the short genome length of phiX174. As a result, a high fraction of the reads cover the genome multiple times. (The genome length is 5.386kb, not 50kb as the reviewer states.)

We do not combine reads because then we would lose information on linkage of mutations across the genomes and on the population fraction of each genotype - both essential for our analysis, and the feature that makes our dataset unique.

See also our response to the question of the reviewer on the average read length in our experiments, and our revised paragraph in lines 98-109.

*Were any of the reads from the PacBio runs bacterial DNA?*

**Response:** We aligned the reads first to the NCBI reference of phiX174, then to the consensus of the ancestor population. The sequencing data we present and analyzed are full-genome length reads of phiX174 only.

We cannot rule out that bacterial DNA was in some of the samples. But the DNA extraction protocol we developed for this study, and which we described in the Materials and Methods, contains several steps reducing bacterial DNA in the samples we sent for sequencing. First, we used a plasmid extraction kit that extracted the phage DNA during the step in its life cyle when it is circularized. (We inhibited protein synthesis in the bacteria by chloramphenicol to prolong the circular phase of the phage life cycle.) Second, we used an Agilent TapeStation with Genomic DNA ScreenTape to select for sequences around 5386 bp of length, and selected against very long DNA sequences - bacterial DNA is likely to be much longer than the phage genome.

After the above mentioned alignment, however, we obtain a dataset (which we refer to as raw data in the paper and shared in the submission) containing only phage genomes.

We are not exactly sure what the reviewer is aiming for with this question. If they insist, we would be happy to align the PacBio reads against the *E. coli* C reference genome, and determine the number of reads associated with the bacterial host.

*L187 "They largely do not overlap with the ancestor population, indicating substantial shifts in the viral composition over time in each evolution line. There is also almost no overlap between the genotype count distributions between the evolution lines. Thus, most genotypes that evolve are new and unique to the evolution line in which they evolved — as would be expected in a regime of neutral evolution." Actually, even in independently evolving replicated population experiencing identical strong selection we do not expect identical genotype distributions - mutation would still introduce randomness. Also, "Unique genotypes" is a term that is frequently used in this study, this is an unusual way to discuss the variation in an evolution experiment. Since these populations are evolving over time, we expect the genotypes to change as populations acquire new mutations or a genotype not being present later in the experiment - even though you observe unique genotypes, these will have arrived from lineages that you observed in earlier time points. Why not consider evolving lineages rather than unique*

**Response:** We thank the reviewer for emphasizing the random nature of evolution. We are certainly aware of that aspect, and it is implemented in our detailed simulations (Material and Methods and Figure 3) as well as the randomization tests that we used to show excess parallel evolution (Materials and Methods and Figure 5). We would like to emphasize, however, that the genotype distributions are far away from identical. Rather, there is almost no overlap (see Figure 3).

Lineages are difficult to reconstruct in evolving populations that are highly diverse, especially if the temporal sampling is sparse (every 200 generations in our experiment). For this reason we focus on new genotypes.

The reviewer highlights a statement we made to describe Figure 4, in which we show the evolution of phiX174 in our experiment on the whole-genome whole-population level. While we admit that some of the aspects we describe could be expected (especially by people who simulate evolution), the more relevant question is whether they have been documented. Has the reviewer seen a experimental documentation of evolution more detailed than what we show in Figure 4 in another paper?

As an aside, we realized that we used “unique genotypes” in two ways: genotypes that have not been observed before, and genotypes that have been observed in only one evolution line (i.e. a non-parallel genotype). (The reviewer is referring to the first use of the term, and seemed to have understood it correctly.) To prevent potential confusion, we now refer to “new” of “distinct” genotypes when referring to genotypes that have not been observed before.

We reserve the attribute “unique” for genotypes that appear in only one evolution line. This use is consistent with how "unique" has been used for mutations in this context for a long time, e. g. in Wichman et al 1999 Science or in Table 2 of Bull et al 1997 Genetics - two studies on phiX174 that we discuss in our paper.

*Line 193 title - this is really confusing. 'Extensive parallel evolution of single mutations, combinations of mutations, and even whole-genome genotypes" If the authors identify genomes from different replicant lineages with identical large sets of mutations how to you rule out cross contamination, isn't that the most likely explanation? Even if there are separating wells etc, what is the probability that 5 mutations co-occur, given that only one or two of them will be beneficial? The simulations show that you only expect to see the parallel evolution of two-variant genotypes by neutral processes. Given that several of the variants are synonymous substitutions, the only answer can be that all five mutations are strongly selected, including the synonymous subs. When the authors do the migration analysis, this just supports that there has been cross contamination. The authors write that "In a nutshell, the migration rate estimates are significantly different from zero, which*

*they should not be because there is no migration between the evolution lines in the experiment." Or… there could be migration. In my opinion is this probably cross contamination - and that doesn't invalidate this experiment or make it less worth reporting, however that authors data and analysis point to contamination, so the most likely answer is that it is migration/cross contamination. Currenly the authors assert that cross contamination is not possible because of the care they took with the experiment - I agree that they have been very careful, but the measures they describe do not rule out the possibility that contamination has happened. I think the authors should either explain what I am missing or acknowledge the possibility of migration/cross contamination explaining the repeated evolution of identical complex genotypes.*

**Response:** Contamination is not the most likely explanation for our results because there are several contradicting patterns in the data. We had addressed this issue in the Result section (lines 197-210), and gave reasons why it is unlikely that the observed parallel evolution is a result of erroneous transfer of virus from one evolution line to another: (i) control lines between the evolution lines, (ii) many instances of parallelism in non-neighboring lines. Additionally, the parallel genotypes are never the most frequent genotypes in any of the evolution lines in which they appeared (as can be seen in Figure 4).

Also, in Figure 6B we show the migration rates estimated without the 26 parallel genotypes. This analysis still shows the bias towards non-zero migration rates. This shows that the bias in the phylodynamic estimates of the migration rates is not primarily driven by the parallel genotypes.

By contamination, it would be genotypes that would be transferred across evolution lines, rather than just mutations. Thus, most of the parallel mutations we observe cannot be arisen by contamination as they do not appear on parallel genotypes. If we delete parallel mutations from the phylodynamic migration rate estimation (as suggested by Reviewer #1), we find that the significant bias disappears (see our response to Reviewer #1 above).

Taken together, this strongly supports that parallel evolution, rather than contamination, explains the bias of the migration rates in the phylodynamic analysis we observe.

But because, generally, contamination can never be completely ruled out we have rephrased the sentence concluding this paragraph (now lines 219-220). Instead of “Thus these genotypes represent instances of parallel evolution”, we now write: “Thus these genotypes most likely represent instances of parallel evolution.

*Figure 4 legend typo - should be "not all genotypes"*

**Response:** Thanks – corrected!

*Line 265 Is the migration assays simply telling the authors that there is migration? How strong would selection have to be to mimic a migration rate of 0.275%?*

**Response:** We lack a formal understanding of the relationship between the nature and strength of selection and the bias in migration rates. We have recently tackled this problem (Ochsner et al *Mol Biol Evol* 2026), but are still only scratching the surface. The main problem is that there are too many selection scenarios to consider: a plethora of various static fitness landscapes with different starting points, then landscapes that vary over time, and lastly dynamical scenarios in which the fitness depends on the other players in the systems, such as specific immune responses.

*Figure 6 legend typo "mugration"*

**Response:** "mugration" is actually a technical term - that is why we italicized it in the main text. It is used to describe a specific migration rate estimation, in which the migration rate is estimated formally as a mutation rate. (This is done by introducing an additional hypothetical locus on the genomes that can have as many alleles as there are locations.) This approach was proposed by Lemey et al 2009 PLOS Comp Biol. We now added this reference after we use the term.

*Line 419 onwards - were the estimates of bacterial counts carried for more than one of the cultures and should have error estimates ie +/- some confidence interval,*

**Response:** We actually determined the CFU listed in that paragraph in only one well each. But we realized that we had wrongly reported the incubation time of the bacteria as 90 minutes. It is actucally 120 minutes at the start of the experiment and appraoximately 135 minutes thereafter. The reason is the automated pipetting regime that had slight variations in length due to a difference tasks being performed (initailly no phage transfer, sometimes sampling). We therefore repeated the measurements of the bacterial densities six times after 120 and 135 minutes, and give standard errors in the revised manuscript. As a result of correcting this mistake, the bascterial desnsities are larger than what we previously reported and as a consequence the MOI is lower, and below 1 throughout the experiment. We revised the paragraph describing the measurements of bacterial densities and the MOI accordingly (lines 464-482).

**Revised text**:

To determine the multiplicity of infection (MOI), the densities of *E. coli C* and phage φX174 were quantified within the experimental wells. Host cell density was determined via standard colony-forming unit (CFU) assays. Briefly, 5 µL of an overnight *E. coli C* culture were inoculated into 200 µL of LB Miller medium per well. We incubated the bacterial populations at 37◦C for 120 and 135 minutes as the duration of the automated pipetting regime was slightly variable over the course of the experiment, and increased from 120 minutes at the start to approximately 135 minutes during the experiment. For each duration, samples were diluted and plated on Hard Agar plates in six replicates to back-calculate the precise density within each well. The bacterial densities were (6.58 ± 2.05) × 10^7^ CFU/well and (7.93 ±2.48) × 10^7^ CFU/well for 120 and 135 minutes of incubation, respectively, corresponding to (3.21 ± 1.00) × 10^8^ CFU/mL and (3.87 ± 1.21) × 10^8^ CFU/mL.

Bacteriophage titers were quantified via plaque-forming unit (PFU) assays at generations 0, 196, and 412. Phage abundances are reported relative to a 4 µL volume, corresponding to the specific initiation and transfer volume propagated between wells each generation. Absolute viral abundances within this reference volume were 1.44 × 10^6^ PFU at generation 0, 2.42 × 10^7^ PFU at generation 196 and 5.12 × 10^7^ PFU at generation 412. Combining these PFU measurements with the number of bacteria per well we obtain MOIs of 0.022, 0.31, and 0.65 at generation 0, 196, and 412, respectively.

*Line 437 Reading through the methods I am struck by the care the authors have taken to develop and validate protocols for phage evolution and especially phage DNA sequencing. Obtaining good DNA sequences for phage is not trivial, and the methods section of this study will be a great resource. I want to check my understanding of the sequencing - it looks like the dsDNA protocol worked, and that this does not have an amplification step. That is best, because as the authors acknowledge in the results the amplification can introduce variants. Back in the results - Line 104, the authors write that: "This raises the question whether they were present in the sample or whether they arose due to mutations introduced during the PCR or cell amplification involved in extracting the DNA and preparing it for sequencing, or due to errors introduced during PacBio sequencing that were not corrected through the circular consensus reads." You can remove PCR as a source of error?*

**Response:** The reviewer is correct that the dsDNA protocol does not involve a PCR amplification step. However, it involves amplifying the phage in cells. While we cannot remove errors that are introduced during the PCR or cell amplification, we can account for them in our simulations. In these simulations, we can let them happen with a certain empirically derived probability and then assess how likely it is that a genotype arose by errors introduced during amplification. We found that three instances or more of a genotype in our raw data make it extrememly unlikely that this genotype arose by procedural error. We then reduce our dataset to only those genotypes that occur three time or more in a sample. This removes many genotypes: the raw data contain 31,229, the reduced dataset only 884 (which is still a lot!).

*You mention that the average read length for PacBio is 25kb - what did you get in your runs? Do the authors think you were capturing an entire single phage in most of the SMRT cells? This would be a good think to mention in the results.*

**Response:** This was a general statement about the PacBio technology at the time when we used it. In our experiment, the actual numbers are even better: if we query the raw data before the circular consensus sequences were derived, the average read length was over 38kb. This does not mean that the majority of reads were larger than 25kb, but close: 229’893 of the total 572’173 reads (40.2%) were longer than 25kb.

This fraction becomes generally larger in the circular consensus sequences as the low quality reads with too short reads (i.e. too few circle passes) are filtered out. And since we have restricted the circular consensus sequences to a quality score of larger than 47 – after all we use only the best 112’734 circular consensus sequences - we are certainly dealing with a majority of sequences in which most phage genomes have been re-sequenced at 5 times or more. A quality score of higher than 47 translates into 7 times or more.

We now mention these specific numbers in the Paragraph in lines 98-109 instead of the generic statement about the average PacBio read length.

**Revised paragraph:**

PacBio sequencing requires double-stranded DNA that is turned into a circular template. Because the average read length of PacBio is several times longer than the genome of φX174 — over 38 kb in our experiment — the majority of reads contain multiple, (re-)sequenced copies of each sampled phage genome. By considering the consistency with which each nucleotide has been called at a given position, one can derive a quality score for each position, and generate the so-called circular consensus sequence (Acevedo et al., 2014, Wenger et al., 2019). Thus, the high error rate of PacBio, and other long-read sequencing platforms, of a single nucleotide read (approximately 10-15%) is substantially reduced by reading each position multiple times (Wenger et al., 2019). To further increase the accuracy of our whole-genome sequences, we restrict the circular consensus sequences to quality scores higher than 47, which translates to a sequencing error rate of 2 × 10^−5^ per nucleotide, or to less than one mis-called nucleotide per genome.

*The abstract states that evolution is "largely neutral," but the manuscript does not provide a clear measure or definition of neutrality. I encourage the authors to include an explicit analysis or justification. The authors also say in the abstract that their parallel evolution analysis is inconsistent with neutral evolution, which the way it is phrased, contradicts their earlier statement about neutrality.*

**Response:** Thanks for pointing this out!

We tried to refrain from characterizing the evolution as neutral in the manuscript - because it is not. We prefer to emphasize that certain patterns of diversifications are either consistent or inconsistent with neutral evolution, for example in line 198-200:

"Thus, most genotypes that evolve are new and unique to the evolution line in which they evolved — as would be expected in a regime of neutral evolution."

The statement in the abstract is a rudiment of earlier versions. We now write:

"... we find that the patterns of viral diversification are largely consistent with neutral evolution, but …"

We would like to emphasize that we apply formal statistical tests against a neutral expectation when establishing excess parallelism of single mutations and their combinations. (See the grey bars in Figure 5).

## Reviewer #3:

*The manuscript by Bons et al. titled " Parallel evolution of full-length genomes in a long-term evolution experiment with phage φX174", describes a series of experiments with the aim of describing the evolution of separate populations of viruses "without imposing any direct selection pressure". The authors find that by using long-read sequencing (PacBio) they can track the sequence variation of entire PhiX174 genomes over 412 generations. They find that evolution is neutral overall but they interestingly find several cases of parallel evolution. The long-read technology the authors use allows them to see parallel evolution at entire genome levels.*

*The manuscript is interesting and ambitious in its aims but I found a number of serious issues that would need to be resolved before the veracity of the paper's claims could be convincing.*

*Major Comments*

*1. The authors describe their experiment as using neutral evolution but it seems from reading the materials and methods that the authors transferred both phage and bacteria during their passaging experiments. No mention of filtering (0.22 um) was made. Thus, rare phage-resistant bacteria would likely have been transferred. Since the hosts were not sequenced as part of the experiments this is hard to assess directly.*

*The authors also acknowledge that bacteria are transferred in the Figure 1 caption and state that "Thus only the bacteriophage populations could evolve during the experiment." This is an untested assumption that is highly likely to be wrong. The growth advantage enjoyed by a resistant bacteria transferred from one well to another during a passage would be significant. Phage-resistant bacterial transfer would in turn result in selection of the PhiX174 population to productively infect these resistant strains and so forth. For example, in my research group I have observed parallel evolution with other phages where we were trying to find variants that could infect a bacterial strain that was phage-resistant. We performed serial passage experiments against these strains several times, months apart, and got exactly the same mutations that enabled the phage to infect this resistant strain. This looks a lot like what the authors observe and attribute to parallel evolution under neutral conditions.*

**Response:** While we appreciate the reviewers focus on the evolution of the bacterial population, this is extremely unlikely in our experimental setting: we transfer 2% of the phages (and some bacteria) every 26 minutes, which corresponds to their lysis time and transfer them into a well that has been seeded with a billion bacteria from a stock. This setting does not allow for the enrichment of a few resistant bacteria within a well - even if their growth advantage is significant - and leads to the resistant bacteria to be washed out fast. If we assume 10 resistant bacteria with **complete** resistance to phage infection and zero cost of resistance, the resistant bacteria disappear at the subsequent transfer.

Simulations based on simple principles of microbial population biology confirm this (see new Figure S1 and the simulation code “Resistance\ simulations.ipynb” that is now included in the supplementary zip-archive “data+code-for-reviewers-May25-2026.zip”). Even if we assume unrealistically high levels of pre-existing resistant bacteria, 30%, resistance bacteria are washed out within less than 4 transfers.

For convenience, we paste the new Figures S1 and its legend (page 46 of the revised manuscript) here:

We would have liked to compare our setting (that clearly does not allow for the enrichment of resistant bacteria) to the experiments of the reviewer. Was the transfer frequency in these experiments equal to the lysis time in their experiments, too? And was the fraction transferred also comparably low (2%)?

*2. Starting on pg. 7, the authors use simulations "In order to quantify the extent to which mutations are introduced during the DNA extraction and preparation" steps. Using another sequencing method (e.g. Nanopore) that does not require a PCR amplification step would have been much more illustrative. PacBio also makes sequencing kits that do not require PCR-amplification. This would not need to be done for the whole experiment, just for a few sets, such as the initial step to quantify this effect.*

**Response:**

In the Materials & Methods section we had stated that “New protocols were needed because PacBio long-read sequencing requires large amounts of phage DNA.” While promotional material from Pacific Biosciences can sound more optimistic generally, and may state specifically that PCR amplification is not needed, it requires 1µg of DNA. (By the way, the main DNA extraction protocol (dsDNA) we used amplified the phage sample in cells, not by PCR.)

To clarify the need of amplification consider the following simple calculation: a base pair weigh approximately 660 Da ≈ 660 g/mol ≈10^-21^g. Because the genome of phiX174 is 5386bp long, this amounts to 6✕10^-18^ g per whole-genome dsDNA. Since there are approximate 5✕10^8^ PFU in the wells during our experiment, the wells contain approximately 0.003 µg of phage dsDNA. This is three orders of magnitude too low for unamplified PacBio sequencing. Factoring in that we sample only about 10% of the phage population in each well, and that extracting phage dsDNA is not perfectly efficient, the need to amplify should be evident.

**Revised Text:**

New protocols were needed because PacBio long-read sequencing requires 1µg of phage DNA, many orders of magnitude more than we have in each well during our experiment.

*3. The authors state that they began the experiment with a "genetically heterogeneous stock population of φX174" but no evidence is shown. It would be quite important to give significantly more detail on this. How do you know it is heterogeneous? What quantitative information can you give about this?*

**Response:** We state repeatedly that the ancestor population consists of 53 genotypes, showed the genotype count distribution in Figure 4 (in grey color), and provide each genotype in the ancestor population and the mutations each genotype carries in the supplementary data. This provides more detail on the ancestor population than most other studies.

*4. The authors give the impression that the approach they are using is novel in that it sequences whole genomes of a virus over serial passaging. Although this is not my area of expertise, I can see references to this type of approach going back a number of years. For example, this approach seems to have been used to study intra-host evolution of hepatitis C and Flock House Virus.*

*https://www.nature.com/articles/s41467-022-28852-1*

*https://youtu.be/IPiteeI-evY*

*https://doi.org/10.1371/journal.ppat.1006365*

*https://doi.org/10.1093/nar/gkab1259*

*If there is something totally new developed in this work it would be helpful to the reader to reference these prior works and describe how the current work is different.*

**Response:** We certainly did not mean to communicate that we are the first to produce whole-genome data, or the first ones to apply long-read high-throughput sequencing technologies to viruses. We wonder how the reviewer got this impression.

What we state clearly though is that the sequencing data that we generated is more fine-grained and larger than what we have seen before: the high number of (genetically linked!) whole-genome sequences (on the order of 10’000) per sample with exceptionally high accuracy (less than 1 mis-called base per genome).

(See also the revised paragraph in lines 98-109 that details how we achieve higher accuracy than many other studies.)

We appreciate the references the reviewer lists. Let us clarify how we go beyond each of the studies mentioned:

1. *https://www.nature.com/articles/s41467-022-28852-1*

*"Rescuing low frequency variants within intra-host viral populations directly from Oxford Nanopore sequencing data"*

*This study has been conducted in the spirit of detecting low frequency variants, and does not aim to give a quantitative picture of the evolution of the entire virus population that we termed "whole-genome whole-population" sequencing.*

*Moreover, they used Oxford Nanopore Technology (ONT), which is associated with a high-error rate. The authors state "However, due to the relatively high error rate of ONT, ranging from 5% to 15%, true variation within hosts is obscured by sequencing errors contained in the raw data." This is many orders of magnitude different to our error rate of 2 × 10^−5^ per nucleotide.*

2. *https://youtu.be/IPiteeI-evY*

*“Resolving Viral Evolution and Quasispecies Diversity with HiFi Sequencing”*

*This is a video promotional event organized by PacBio high-lighting different uses of Pacbio HiFi sequencing (which has been described in a 2019 paper by Wenger et al that we had cited).*

*The examples are either not whole-genome, or do not compare in terms of the numbers of sequences generated to our study. This applies to the studies mentioned by Meredith Ashby (PacBio) as well as to the study presented by the invited speaker, Eli Meritz (NIH).*

*Eli Meritz specifically states they can generate accurate sequences “of hundreds to thousands of individual virus genomes” per sample (timestamp 32:32), which is an order of magnitude lower than what we have per sample.*

*The figure that Meredith Ashby shows (timestamp 3:09) comparing the quality of sequencing quality of ONT Illumina and PacBio HiFi is particularly noteworthy: PacBio HiFi generate sequences with a quality score of approximately 33, a little higher than Illumina but with higher depths and read length. By restricting our sequences to a quality score of over 47, we clearly go beyond the standard use of PacBio HiFi.*

3. [*https://doi.org/10.1371/journal.ppat.1006365*](https://doi.org/10.1371/journal.ppat.1006365)

*"Parallel ClickSeq and Nanopore sequencing elucidates the rapid evolution of defective-interfering RNAs in Flock House virus"*

*In this study, the authors present similarly high numbers of sequence: "We obtained a total of 169’814 reads, of which 46’183 passed the default ONT filter and were successfully demultiplexed. This yielded between 2688 and 8815 full-length reads per passage and corresponds to approximately 0.1 Gigabases of sequence information. "*

*But since they used the ONT platform for their full-length genome sequencing, they have an error rate of 7% per nucleotide, which is, again, many orders of magnitude different to our error rate of 2 × 10^−5^ per nucleotide.*

4. *https://doi.org/10.1093/nar/gkab1259*

*"Covariation of viral recombination with single nucleotide variants during virus evolution revealed by CoVaM"*

*Also this paper used the ONT and hence generated sequences with a 7% error rate.*

If the reviewer knows a paper that presents such data please tell us. We have been at this for years and have been searching for precedents but could not find any. We have also been speaking about these data at international conferences (such as the Meeting of the International Society for Evolution, Medicine and Public Health, the Conference Jacques Monod on Virus Evolution, and the Workshop on Virus Dynamics) with many experts present and were not alerted to any comparable dataset.
