# Supplementary material for: Full-length genomes evolve in parallel in a long-term evolution experiment with phage φX174
Source: PLoS Biol. 2026 Oct 29;24(10):e3004064. doi: 10.1371/journal.pbio.3004064 (PMC13743035; doi:10.1371/journal.pbio.3004064)
Supplement: Peer review history

# Editor report: Decision Letter 0

**DOI:** 10.1371/journal.pbio.3004064.r001

**Authors:** Roland G Roberts

7 Aug 2025

Dear Roland,

Thank you for submitting your manuscript entitled "Parallel evolution of full-length genomes in a long-term evolution experiment with phage phiX174" for consideration as a Research Article by PLOS Biology.

Your manuscript has now been evaluated by the PLOS Biology editorial staff, as well as by an academic editor with relevant expertise, and I'm writing to let you know that we would like to send your submission out for external peer review.

However, before we can send your manuscript to reviewers, we need you to complete your submission by providing the metadata that is required for full assessment. To this end, please login to Editorial Manager where you will find the paper in the 'Submissions Needing Revisions' folder on your homepage. Please click 'Revise Submission' from the Action Links and complete all additional questions in the submission questionnaire.

Once your full submission is complete, your paper will undergo a series of checks in preparation for peer review. After your manuscript has passed the checks it will be sent out for review. To provide the metadata for your submission, please Login to Editorial Manager (https://www.editorialmanager.com/pbiology) within two working days, i.e. by Aug 11 2025 11:59PM.

If your manuscript has been previously peer-reviewed at another journal, PLOS Biology is willing to work with those reviews in order to avoid re-starting the process. Submission of the previous reviews is entirely optional and our ability to use them effectively will depend on the willingness of the previous journal to confirm the content of the reports and share the reviewer identities. Please note that we reserve the right to invite additional reviewers if we consider that additional/independent reviewers are needed, although we aim to avoid this as far as possible. In our experience, working with previous reviews does save time.

If you would like us to consider previous reviewer reports, please edit your cover letter to let us know and include the name of the journal where the work was previously considered and the manuscript ID it was given. In addition, please upload a response to the reviews as a 'Prior Peer Review' file type, which should include the reports in full and a point-by-point reply detailing how you have or plan to address the reviewers' concerns.

During the process of completing your manuscript submission, you will be invited to opt-in to posting your pre-review manuscript as a bioRxiv preprint. Visit http://journals.plos.org/plosbiology/s/preprints for full details. If you consent to posting your current manuscript as a preprint, please upload a single Preprint PDF.

Feel free to email us at plosbiology@plos.org if you have any queries relating to your submission.

Kind regards,

Roli

Roland Roberts, PhD

Senior Editor

PLOS Biology

rroberts@plos.org

# Peer review: Decision Letter 1

**DOI:** 10.1371/journal.pbio.3004064.r002

**Authors:** Roland G Roberts

26 Sep 2025

Dear Roland,

Thank you for your patience while your manuscript "Parallel evolution of full-length genomes in a long-term evolution experiment with phage phiX174" was peer-reviewed by PLOS Biology. Your work was assessed and discussed by the PLOS Biology editorial team, an Academic Editor with relevant expertise, and by three independent reviewers. Based on the reviews, which you will find at the end of this email, I regret that we will not be pursuing your manuscript for publication in the journal.

You'll see that reviewer #1 is very positive, but suggests several analyses to better support the claims. Reviewer #2 liked the experiment, but raises some questions about the analysis and the support for the claims; importantly, s/he ends up questioning whether the experiment is really neutral. Reviewer #3 calls the study “interesting and ambitious,” but has some serious concerns, in particular about the neutrality of the experiment, especially given that bacteria are transferred with the phage, and resistant bacteria would exert strong selective pressure on the phage (s/he suggests that you could sequence the bacteria to show that they are unchanged). S/he also suggests non-amplification-dependent sequencing, evidence for initial phage heterogeneity, and wonders about the novelty of the approach.

I discussed these comments, and particularly the shared concerns from reviewers #2 and #3 about the neutrality of the experiment. They said, "I think it is clear from the reviewers comments that there is a major flaw in the experimental design, and as #R3 clearly articulates in their cross-examination, the fact that bacteria were also transferred, I would also be confident in assuming that phage-resistant variants were selected for and transferred across the experimental design. This does have major implications for the authors claims the novelty of the manuscript."

I'm sorry that we can't be more positive on this occasion and hope the reviewer reports will help you in preparing your manuscript for submission elsewhere.

While we cannot consider your manuscript for publication in PLOS Biology, we suggest that you consider transferring your manuscript to PLOS Genetics. However, please note that the PLOS journals are editorially independent, so we cannot guarantee it will be reviewed there.

If you would like to submit your work to PLOS Genetics, please click the following link:

<DeepLinkData><DeepLinkTypeID>27</DeepLinkTypeID><peopleID>62739</peopleID><userSecurityID>e31bc7e9-a91a-4aab-9c85-c698c4c5a113</userSecurityID><documentID>64942</documentID><revision>1</revision><manuscriptNumber>PBIOLOGY-D-25-02443</manuscriptNumber><docSecurityID>14f0a6db-1551-4ad8-8a4e-92d1c2833652</docSecurityID></DeepLinkData>

If you do not wish to submit your work to PLOS Genetics, please click this link to decline:

<DeepLinkData><DeepLinkTypeID>28</DeepLinkTypeID><peopleID>62739</peopleID><userSecurityID>e31bc7e9-a91a-4aab-9c85-c698c4c5a113</userSecurityID><documentID>64942</documentID><revision>1</revision><manuscriptNumber>PBIOLOGY-D-25-02443</manuscriptNumber><docSecurityID>14f0a6db-1551-4ad8-8a4e-92d1c2833652</docSecurityID></DeepLinkData>

Please note, you can log into the submission sites with the same login that you used to submit to this journal.

Should you choose to transfer your submission, you will receive a confirmation email within 24-48 hours after accepting the transfer. If you have any questions, please feel free to contact the journal at plosbiology@plos.org.

I hope you understand the reasons for this decision and that you will consider publishing your work in PLOS in future. Thank you for your support of PLOS and of Open Access publishing.

Sincerely,

Roli

Roland Roberts, PhD

Senior Editor

PLOS Biology

rroberts@plos.org

------------------------------------------------------------------------

REVIEWERS' COMMENTS:

Reviewer #1:

[identifies himself as David A. Rasmussen]

Bons el. combine experimental evolution of the phage phiX1743 with long-read (whole genome) sequencing. These methods allow the authors to track the evolutionary dynamics of full-length haplotypes in the viral population, which few viral deep sequencing studies have done before. One key result is that there is a high degree of convergent evolution across multiple sites in the genome. While such convergent evolution in phage evolution studies has been reported before (Bull et al., 1997), its particularly surprising here as the phage populations were not subjected to any novel selection pressure, host or environment. The extent of convergent evolution has major implications for reconstructing viral phylogenies accurately, and therefore downstream inferences from drawn from these phylogenies.

Overall, the work reported here is of high quality and I only have a few minor comments where certain arguments could be better supported by additional analysis. While I don't want to dictate how the authors present their work, I also thought that the emphasis on migration rates biases somewhat misses the larger story here about how convergent evolution could bias phylogenetic inference more generally.

***************

Major comments:

1) Accuracy of phylogenetic reconstructions: Presumably migration rates are estimated to be non-zero in the phylodynamic analyses because samples from different evolutionary lines are interspersed with one another in the reconstructed trees. This suggests there is a bigger underlying problem with phylogenetic inference in the presence of convergent evolution, consistent with the problem of "exceptional convergent evolution" identified by Bull et al. (1997). This to me is the bigger story here since nearly all downstream phylodynamic and demographic inference methods assume that the trees can be reconstructed reasonably accuracy. It might therefore be interesting to quantify the degree of phylogenetic error caused by convergent evolution. This seems fairly straightforward in this case as you know there's a reconstruction error any time samples from the same evolutionary lineage fall outside the same clade, such that the extent of polyphyletic relationships among samples from the same line can be used to quantify the extent of phylogenetic error.

2) Source of phylogenetic error: It has become standard practice in some fields to mask or remove sites undergoing convergent evolution from alignments prior to phylogenetic analysis i.e. sites conferring drug resistance mutations in viral genomes. In order to more definitively show that convergent evolution is the source of phylogenetic error and biases in migration rates, it might therefore be interesting to mask the sites undergoing convergent evolution in the alignment. Showing that masking these sites eliminates the upwards bias in migration rates would provide strong support that it is actually parallel evolution driving this bias.

3) Fitness of genotypes: The authors argue that the mutations that repeatedly reoccur in the same haplotype are likely positively epistatic. This claim seems like it could be better supported. One could estimate the relative fitness (selection coefficients) acting on both single and double mutants (e.g. by using genetic time series methods) to show that the selective advantage of the double mutants is more than the additive effects of the single mutants.

***************

Minor comments:

Line 44-45: "phylodynamic inference methods have not yet been experimentally assessed." -- One could argue that they have been extensively experimentally assessed by simulation, just not with carefully controlled in vivo experiments.

Line 52-53: "high-throughput sequencing can detect low frequency mutations, but at the cost of loosing linkage information" -- I don't think this is inherent to HTS, but specific short-read sequencing technologies. Long read platforms would not suffer from the same constraints on assembling haplotypes.

This might just be an issue of semantics, but it sounds odd to claim these populations are evolving without selection pressures since even in a stable environment any phage population would be under very strong selection to survive, replicate, compete for hosts, and so on. Maybe it would be more clear to say that these populations were evolved in the absence of "novel selection pressures"?

Reviewer #2:

In this study, the authors performed an interesting evolution experiment using bacteriophage and bacterial hosts in a highly novel design. They transferred phage populations onto naive bacteria every 26 minutes across 412 sequential transfers, using a robotic liquid handler. They complemented this experiment with extensive high-quality DNA sequencing and simulations to report on the evolutionary dynamics of evolution in replicate phage populations. This approach represents a new and interesting direction for experimental evolution and has generated a valuable dataset. While the work is innovative and the data are impressive, I have questions primarily about the analysis and the strength of some of the conclusions.

Comments:

Line 34 Variant should be variants.

L49 "On the one hand"

L60 sentence requires a period.

L84 perhaps "the rationale for not intentionally applying selection" would be clearer?

L102 The authors write that "We obtain over 110'000 reads covering the entire genome of φX174, representing" over 31'000 unique genotypes" Is this evenly distributed across the 8 populations you sequenced? (4 replicates x 2 time points)? I think the authors should report the coverage per replicate population and time point. If the coverage per replicate population is approximately equal, then you are getting at least 10,000 reads per population. Since your maximum read length is 5kb (the length of the phage genome), this puts you at around 10000x coverage for the population. What is your Qscore for each of your genomes? If it is Q30, we expect 5 errors for each 50kb genome - I believe Qscore for PacBio is incorporates the benefits of the smrt cell repeated rolling circle sequencing approach. This means many errors will be included in your analysis. Why not combine reads from multiple smrt cells to so that you can get the benefits of high coverage?

Were any of the reads from the PacBio runs bacterial DNA?

L187 "They largely do not overlap with the ancestor population, indicating substantial shifts in the viral composition over time in each evolution line. There is also almost no overlap between the genotype count distributions between the evolution lines. Thus, most genotypes that evolve are new and unique to the evolution line in which they evolved — as would be expected in a regime of neutral evolution." Actually, even in independently evolving replicated population experiencing identical strong selection we do not expect identical genotype distributions - mutation would still introduce randomness. Also, "Unique genotypes" is a term that is frequently used in this study, this is an unusual way to discuss the variation in an evolution experiment. Since these populations are evolving over time, we expect the genotypes to change as populations acquire new mutations or a genotype not being present later in the experiment - even though you observe unique genotypes, these will have arrived from lineages that you observed in earlier time points. Why not consider evolving lineages rather than unique

Line 193 title - this is really confusing. 'Extensive parallel evolution of single mutations, combinations of mutations, and even whole-genome genotypes" If the authors identify genomes from different replicant lineages with identical large sets of mutations how to you rule out cross contamination, isn't that the most likely explanation? Even if there are separating wells etc, what is the probability that 5 mutations co-occur, given that only one or two of them will be beneficial? The simulations show that you only expect to see the parallel evolution of two-variant genotypes by neutral processes. Given that several of the variants are synonymous substitutions, the only answer can be that all five mutations are strongly selected, including the synonymous subs. When the authors do the migration analysis, this just supports that there has been cross contamination. The authors write that "In a nutshell, the migration rate estimates are significantly different from zero, which they should not be because there is no migration between the evolution lines in the experiment." Or… there could be migration. In my opinion is this probably cross contamination - and that doesn't invalidate this experiment or make it less worth reporting, however that authors data and analysis point to contamination, so the most likely answer is that it is migration/cross contamination. Currenly the authors assert that cross contamination is not possible because of the care they took with the experiment - I agree that they have been very careful, but the measures they describe do not rule out the possibility that contamination has happened. I think the authors should either explain what I am missing or acknowledge the possibility of migration/cross contamination explaining the repeated evolution of identical complex genotypes.

Figure 4 legend typo - should be "not all genotypes"

Line 265 Is the migration assays simply telling the authors that there is migration? How strong would selection have to be to mimic a migration rate of 0.275%?

Figure 6 legend typo "mugration"

Line 419 onwards - were the estimates of bacterial counts carried for more than one of the cultures and should have error estimates ie +/- some confidence interval,

Line 437 Reading through the methods I am struck by the care the authors have taken to develop and validate protocols for phage evolution and especially phage DNA sequencing. Obtaining good DNA sequences for phage is not trivial, and the methods section of this study will be a great resource. I want to check my understanding of the sequencing - it looks like the dsDNA protocol worked, and that this does not have an amplification step. That is best, because as the authors acknowledge in the results the amplification can introduce variants. Back in the results - Line 104, the authors write that: "This raises the question whether they were present in the sample or whether they arose due to mutations introduced during the PCR or cell amplification involved in extracting the DNA and preparing it for sequencing, or due to errors introduced during PacBio sequencing that were not corrected through the circular consensus reads." You can remove PCR as a source of error?

You mention that the average read length for PacBio is 25kb - what did you get in your runs? Do the authors think you were capturing an entire single phage in most of the SMRT cells? This would be a good think to mention in the results.

The abstract states that evolution is "largely neutral," but the manuscript does not provide a clear measure or definition of neutrality. I encourage the authors to include an explicit analysis or justification. The authors also say in the abstract that their parallel evolution analysis is inconsistent with neutral evolution, which the way it is phrased, contradicts their earlier statement about neutrality.

Reviewer #3:

The manuscript by Bons et al. titled " Parallel evolution of full-length genomes in a long-term evolution experiment with phage φX174", describes a series of experiments with the aim of describing the evolution of separate populations of viruses "without imposing any direct selection pressure". The authors find that by using long-read sequencing (PacBio) they can track the sequence variation of entire PhiX174 genomes over 412 generations. They find that evolution is neutral overall but they interestingly find several cases of parallel evolution. The long-read technology the authors use allows them to see parallel evolution at entire genome levels.

The manuscript is interesting and ambitious in its aims but I found a number of serious issues that would need to be resolved before the veracity of the paper's claims could be convincing.

Major Comments

1. The authors describe their experiment as using neutral evolution but it seems from reading the materials and methods that the authors transferred both phage and bacteria during their passaging experiments. No mention of filtering (0.22 um) was made. Thus, rare phage-resistant bacteria would likely have been transferred. Since the hosts were not sequenced as part of the experiments this is hard to assess directly.

The authors also acknowledge that bacteria are transferred in the Figure 1 caption and state that "Thus only the bacteriophage populations could evolve during the experiment." This is an untested assumption that is highly likely to be wrong. The growth advantage enjoyed by a resistant bacteria transferred from one well to another during a passage would be significant. Phage-resistant bacterial transfer would in turn result in selection of the PhiX174 population to productively infect these resistant strains and so forth. For example, in my research group I have observed parallel evolution with other phages where we were trying to find variants that could infect a bacterial strain that was phage-resistant. We performed serial passage experiments against these strains several times, months apart, and got exactly the same mutations that enabled the phage to infect this resistant strain. This looks a lot like what the authors observe and attribute to parallel evolution under neutral conditions.

2. Starting on pg. 7, the authors use simulations "In order to quantify the extent to which mutations are introduced during the DNA extraction and preparation" steps. Using another sequencing method (e.g. Nanopore) that does not require a PCR amplification step would have been much more illustrative. PacBio also makes sequencing kits that do not require PCR-amplification. This would not need to be done for the whole experiment, just for a few sets, such as the initial step to quantify this effect.

3. The authors state that they began the experiment with a "genetically heterogeneous stock population of φX174" but no evidence is shown. It would be quite important to give significantly more detail on this. How do you know it is heterogeneous? What quantitative information can you give about this?

4. The authors give the impression that the approach they are using is novel in that it sequences whole genomes of a virus over serial passaging. Although this is not my area of expertise, I can see references to this type of approach going back a number of years. For example, this approach seems to have been used to study intra-host evolution of hepatitis C and Flock House Virus.

https://www.nature.com/articles/s41467-022-28852-1

https://youtu.be/IPiteeI-evY

https://doi.org/10.1371/journal.ppat.1006365

https://doi.org/10.1093/nar/gkab1259

If there is something totally new developed in this work it would be helpful to the reader to reference these prior works and describe how the current work is different.

# Editor report: Decision Letter 2

**DOI:** 10.1371/journal.pbio.3004064.r003

**Authors:** Roland G Roberts

14 May 2026

Dear Roland,

Thank you for your patience while we considered your Appeal regarding the previous decision on your manuscript "Parallel evolution of full-length genomes in a long-term evolution experiment with phage phiX174." The points that you raised have now been evaluated by the PLOS Biology editors and the Academic Editor, and in the light of these discussions, we would like to give you the opportunity to revise the work to thoroughly address the reviewers' reports.

As previously mentioned, you'll see that reviewer #1 is very positive, but suggests several analyses to better support the claims. Reviewer #2 liked the experiment, but raises some questions about the analysis and the support for the claims; importantly, s/he ends up questioning whether the experiment is really neutral. Reviewer #3 calls the study “interesting and ambitious,” but has some serious concerns, in particular about the neutrality of the experiment, especially given that bacteria are transferred with the phage, and resistant bacteria would exert strong selective pressure on the phage (s/he suggests that you could sequence the bacteria to show that they are unchanged). S/he also suggests non-amplification-dependent sequencing, evidence for initial phage heterogeneity, and wonders about the novelty of the approach.

We understand from your Appeal that you are in a position to address all of the concerns raised, some of which may have arisen from misunderstandings about the experimental design. Given the extent of revision needed, we cannot make a decision about publication until we have seen the revised manuscript and your response to the reviewers' comments. Your revised manuscript is likely to be sent for further evaluation by all or a subset of the reviewers.

In addition to these revisions, you will need to complete some formatting changes, which you will receive in a follow up email. A member of our team will be in touch with a set of requests shortly.

We expect to receive your revised manuscript within 3 months. Please email us (plosbiology@plos.org) if you have any questions or concerns, or would like to request an extension.

At this stage, your manuscript remains formally under active consideration at our journal; please notify us by email if you do not intend to submit a revision so that we may withdraw it.

**IMPORTANT - SUBMITTING YOUR REVISION**

Your revisions should address the specific points made by each reviewer. Please submit the following files along with your revised manuscript:

1. A 'Response to Reviewers' file - this should detail your responses to the editorial requests, present a point-by-point response to all of the reviewers' comments, and indicate the changes made to the manuscript.

*NOTE: In your point-by-point response to the reviewers, please provide the full context of each review. Do not selectively quote paragraphs or sentences to reply to. The entire set of reviewer comments should be present in full and each specific point should be responded to individually, point by point.

You should also cite any additional relevant literature that has been published since the original submission and mention any additional citations in your response.

2. In addition to a clean copy of the manuscript, please also upload a 'track-changes' version of your manuscript that specifies the edits made. This should be uploaded as a "Revised Article with Changes Highlighted" file type.

*Re-submission Checklist*

When you are ready to resubmit your revised manuscript, please refer to this re-submission checklist: https://plos.io/Biology_Checklist

To submit a revised version of your manuscript, please go to https://www.editorialmanager.com/pbiology/ and log in as an Author. Click the link labelled 'Submissions Needing Revision' where you will find your submission record.

Please make sure to read the following important policies and guidelines while preparing your revision:

*Published Peer Review*

Please note while forming your response, if your article is accepted, you may have the opportunity to make the peer review history publicly available. The record will include editor decision letters (with reviews) and your responses to reviewer comments. If eligible, we will contact you to opt in or out. Please see here for more details:

https://blogs.plos.org/plos/2019/05/plos-journals-now-open-for-published-peer-review/

*PLOS Data Policy*

Please note that as a condition of publication PLOS' data policy (http://journals.plos.org/plosbiology/s/data-availability) requires that you make available all data used to draw the conclusions arrived at in your manuscript. If you have not already done so, you must include any data used in your manuscript either in appropriate repositories, within the body of the manuscript, or as supporting information (N.B. this includes any numerical values that were used to generate graphs, histograms etc.). For an example see here: http://www.plosbiology.org/article/info%3Adoi%2F10.1371%2Fjournal.pbio.1001908#s5

*Blot and Gel Data Policy*

We require the original, uncropped and minimally adjusted images supporting all blot and gel results reported in an article's figures or Supporting Information files. We will require these files before a manuscript can be accepted so please prepare them now, if you have not already uploaded them. Please carefully read our guidelines for how to prepare and upload this data: https://journals.plos.org/plosbiology/s/figures#loc-blot-and-gel-reporting-requirements

*Protocols deposition*

To enhance the reproducibility of your results, we recommend that if applicable you deposit your laboratory protocols in protocols.io, where a protocol can be assigned its own identifier (DOI) such that it can be cited independently in the future. Additionally, PLOS ONE offers an option for publishing peer-reviewed Lab Protocol articles, which describe protocols hosted on protocols.io. Read more information on sharing protocols at https://plos.org/protocols?utm_medium=editorial-email&utm_source=authorletters&utm_campaign=protocols

Thank you again for your submission to our journal. We hope that our editorial process has been constructive thus far, and we welcome your feedback at any time. Please don't hesitate to contact us if you have any questions or comments.

Sincerely,

Roli

Roland Roberts, PhD

Senior Editor

PLOS Biology

rroberts@plos.org

------------------------------------

REVIEWERS' COMMENTS:

Reviewer #1:

[identifies himself as David A. Rasmussen]

Bons el. combine experimental evolution of the phage phiX1743 with long-read (whole genome) sequencing. These methods allow the authors to track the evolutionary dynamics of full-length haplotypes in the viral population, which few viral deep sequencing studies have done before. One key result is that there is a high degree of convergent evolution across multiple sites in the genome. While such convergent evolution in phage evolution studies has been reported before (Bull et al., 1997), its particularly surprising here as the phage populations were not subjected to any novel selection pressure, host or environment. The extent of convergent evolution has major implications for reconstructing viral phylogenies accurately, and therefore downstream inferences from drawn from these phylogenies.

Overall, the work reported here is of high quality and I only have a few minor comments where certain arguments could be better supported by additional analysis. While I don't want to dictate how the authors present their work, I also thought that the emphasis on migration rates biases somewhat misses the larger story here about how convergent evolution could bias phylogenetic inference more generally.

***************

Major comments:

1) Accuracy of phylogenetic reconstructions: Presumably migration rates are estimated to be non-zero in the phylodynamic analyses because samples from different evolutionary lines are interspersed with one another in the reconstructed trees. This suggests there is a bigger underlying problem with phylogenetic inference in the presence of convergent evolution, consistent with the problem of "exceptional convergent evolution" identified by Bull et al. (1997). This to me is the bigger story here since nearly all downstream phylodynamic and demographic inference methods assume that the trees can be reconstructed reasonably accuracy. It might therefore be interesting to quantify the degree of phylogenetic error caused by convergent evolution. This seems fairly straightforward in this case as you know there's a reconstruction error any time samples from the same evolutionary lineage fall outside the same clade, such that the extent of polyphyletic relationships among samples from the same line can be used to quantify the extent of phylogenetic error.

2) Source of phylogenetic error: It has become standard practice in some fields to mask or remove sites undergoing convergent evolution from alignments prior to phylogenetic analysis i.e. sites conferring drug resistance mutations in viral genomes. In order to more definitively show that convergent evolution is the source of phylogenetic error and biases in migration rates, it might therefore be interesting to mask the sites undergoing convergent evolution in the alignment. Showing that masking these sites eliminates the upwards bias in migration rates would provide strong support that it is actually parallel evolution driving this bias.

3) Fitness of genotypes: The authors argue that the mutations that repeatedly reoccur in the same haplotype are likely positively epistatic. This claim seems like it could be better supported. One could estimate the relative fitness (selection coefficients) acting on both single and double mutants (e.g. by using genetic time series methods) to show that the selective advantage of the double mutants is more than the additive effects of the single mutants.

***************

Minor comments:

Line 44-45: "phylodynamic inference methods have not yet been experimentally assessed." -- One could argue that they have been extensively experimentally assessed by simulation, just not with carefully controlled in vivo experiments.

Line 52-53: "high-throughput sequencing can detect low frequency mutations, but at the cost of loosing linkage information" -- I don't think this is inherent to HTS, but specific short-read sequencing technologies. Long read platforms would not suffer from the same constraints on assembling haplotypes.

This might just be an issue of semantics, but it sounds odd to claim these populations are evolving without selection pressures since even in a stable environment any phage population would be under very strong selection to survive, replicate, compete for hosts, and so on. Maybe it would be more clear to say that these populations were evolved in the absence of "novel selection pressures"?

Reviewer #2:

In this study, the authors performed an interesting evolution experiment using bacteriophage and bacterial hosts in a highly novel design. They transferred phage populations onto naive bacteria every 26 minutes across 412 sequential transfers, using a robotic liquid handler. They complemented this experiment with extensive high-quality DNA sequencing and simulations to report on the evolutionary dynamics of evolution in replicate phage populations. This approach represents a new and interesting direction for experimental evolution and has generated a valuable dataset. While the work is innovative and the data are impressive, I have questions primarily about the analysis and the strength of some of the conclusions.

Comments:

Line 34 Variant should be variants.

L49 "On the one hand"

L60 sentence requires a period.

L84 perhaps "the rationale for not intentionally applying selection" would be clearer?

L102 The authors write that "We obtain over 110'000 reads covering the entire genome of φX174, representing" over 31'000 unique genotypes" Is this evenly distributed across the 8 populations you sequenced? (4 replicates x 2 time points)? I think the authors should report the coverage per replicate population and time point. If the coverage per replicate population is approximately equal, then you are getting at least 10,000 reads per population. Since your maximum read length is 5kb (the length of the phage genome), this puts you at around 10000x coverage for the population. What is your Qscore for each of your genomes? If it is Q30, we expect 5 errors for each 50kb genome - I believe Qscore for PacBio is incorporates the benefits of the smrt cell repeated rolling circle sequencing approach. This means many errors will be included in your analysis. Why not combine reads from multiple smrt cells to so that you can get the benefits of high coverage?

Were any of the reads from the PacBio runs bacterial DNA?

L187 "They largely do not overlap with the ancestor population, indicating substantial shifts in the viral composition over time in each evolution line. There is also almost no overlap between the genotype count distributions between the evolution lines. Thus, most genotypes that evolve are new and unique to the evolution line in which they evolved — as would be expected in a regime of neutral evolution." Actually, even in independently evolving replicated population experiencing identical strong selection we do not expect identical genotype distributions - mutation would still introduce randomness. Also, "Unique genotypes" is a term that is frequently used in this study, this is an unusual way to discuss the variation in an evolution experiment. Since these populations are evolving over time, we expect the genotypes to change as populations acquire new mutations or a genotype not being present later in the experiment - even though you observe unique genotypes, these will have arrived from lineages that you observed in earlier time points. Why not consider evolving lineages rather than unique

Line 193 title - this is really confusing. 'Extensive parallel evolution of single mutations, combinations of mutations, and even whole-genome genotypes" If the authors identify genomes from different replicant lineages with identical large sets of mutations how to you rule out cross contamination, isn't that the most likely explanation? Even if there are separating wells etc, what is the probability that 5 mutations co-occur, given that only one or two of them will be beneficial? The simulations show that you only expect to see the parallel evolution of two-variant genotypes by neutral processes. Given that several of the variants are synonymous substitutions, the only answer can be that all five mutations are strongly selected, including the synonymous subs. When the authors do the migration analysis, this just supports that there has been cross contamination. The authors write that "In a nutshell, the migration rate estimates are significantly different from zero, which they should not be because there is no migration between the evolution lines in the experiment." Or… there could be migration. In my opinion is this probably cross contamination - and that doesn't invalidate this experiment or make it less worth reporting, however that authors data and analysis point to contamination, so the most likely answer is that it is migration/cross contamination. Currenly the authors assert that cross contamination is not possible because of the care they took with the experiment - I agree that they have been very careful, but the measures they describe do not rule out the possibility that contamination has happened. I think the authors should either explain what I am missing or acknowledge the possibility of migration/cross contamination explaining the repeated evolution of identical complex genotypes.

Figure 4 legend typo - should be "not all genotypes"

Line 265 Is the migration assays simply telling the authors that there is migration? How strong would selection have to be to mimic a migration rate of 0.275%?

Figure 6 legend typo "mugration"

Line 419 onwards - were the estimates of bacterial counts carried for more than one of the cultures and should have error estimates ie +/- some confidence interval,

Line 437 Reading through the methods I am struck by the care the authors have taken to develop and validate protocols for phage evolution and especially phage DNA sequencing. Obtaining good DNA sequences for phage is not trivial, and the methods section of this study will be a great resource. I want to check my understanding of the sequencing - it looks like the dsDNA protocol worked, and that this does not have an amplification step. That is best, because as the authors acknowledge in the results the amplification can introduce variants. Back in the results - Line 104, the authors write that: "This raises the question whether they were present in the sample or whether they arose due to mutations introduced during the PCR or cell amplification involved in extracting the DNA and preparing it for sequencing, or due to errors introduced during PacBio sequencing that were not corrected through the circular consensus reads." You can remove PCR as a source of error?

You mention that the average read length for PacBio is 25kb - what did you get in your runs? Do the authors think you were capturing an entire single phage in most of the SMRT cells? This would be a good think to mention in the results.

The abstract states that evolution is "largely neutral," but the manuscript does not provide a clear measure or definition of neutrality. I encourage the authors to include an explicit analysis or justification. The authors also say in the abstract that their parallel evolution analysis is inconsistent with neutral evolution, which the way it is phrased, contradicts their earlier statement about neutrality.

Reviewer #3:

The manuscript by Bons et al. titled " Parallel evolution of full-length genomes in a long-term evolution experiment with phage φX174", describes a series of experiments with the aim of describing the evolution of separate populations of viruses "without imposing any direct selection pressure". The authors find that by using long-read sequencing (PacBio) they can track the sequence variation of entire PhiX174 genomes over 412 generations. They find that evolution is neutral overall but they interestingly find several cases of parallel evolution. The long-read technology the authors use allows them to see parallel evolution at entire genome levels.

The manuscript is interesting and ambitious in its aims but I found a number of serious issues that would need to be resolved before the veracity of the paper's claims could be convincing.

Major Comments

1. The authors describe their experiment as using neutral evolution but it seems from reading the materials and methods that the authors transferred both phage and bacteria during their passaging experiments. No mention of filtering (0.22 um) was made. Thus, rare phage-resistant bacteria would likely have been transferred. Since the hosts were not sequenced as part of the experiments this is hard to assess directly.

The authors also acknowledge that bacteria are transferred in the Figure 1 caption and state that "Thus only the bacteriophage populations could evolve during the experiment." This is an untested assumption that is highly likely to be wrong. The growth advantage enjoyed by a resistant bacteria transferred from one well to another during a passage would be significant. Phage-resistant bacterial transfer would in turn result in selection of the PhiX174 population to productively infect these resistant strains and so forth. For example, in my research group I have observed parallel evolution with other phages where we were trying to find variants that could infect a bacterial strain that was phage-resistant. We performed serial passage experiments against these strains several times, months apart, and got exactly the same mutations that enabled the phage to infect this resistant strain. This looks a lot like what the authors observe and attribute to parallel evolution under neutral conditions.

2. Starting on pg. 7, the authors use simulations "In order to quantify the extent to which mutations are introduced during the DNA extraction and preparation" steps. Using another sequencing method (e.g. Nanopore) that does not require a PCR amplification step would have been much more illustrative. PacBio also makes sequencing kits that do not require PCR-amplification. This would not need to be done for the whole experiment, just for a few sets, such as the initial step to quantify this effect.

3. The authors state that they began the experiment with a "genetically heterogeneous stock population of φX174" but no evidence is shown. It would be quite important to give significantly more detail on this. How do you know it is heterogeneous? What quantitative information can you give about this?

4. The authors give the impression that the approach they are using is novel in that it sequences whole genomes of a virus over serial passaging. Although this is not my area of expertise, I can see references to this type of approach going back a number of years. For example, this approach seems to have been used to study intra-host evolution of hepatitis C and Flock House Virus.

https://www.nature.com/articles/s41467-022-28852-1

https://youtu.be/IPiteeI-evY

https://doi.org/10.1371/journal.ppat.1006365

https://doi.org/10.1093/nar/gkab1259

If there is something totally new developed in this work it would be helpful to the reader to reference these prior works and describe how the current work is different.

# Author comment: Author response to Decision Letter 3

**DOI:** 10.1371/journal.pbio.3004064.r004

9 Jul 2026

Attachment Submitted filename: responses-to-reviewers-PB-rev.docx

# Peer review: Decision Letter 3

**DOI:** 10.1371/journal.pbio.3004064.r005

**Authors:** Roland G Roberts

7 Aug 2026

Dear Roland,

Thank you for your patience while we considered your revised manuscript "Parallel evolution of full-length genomes in a long-term evolution experiment with phage phiX174" for consideration as a Research Article at PLOS Biology. Your revised study has now been evaluated by the PLOS Biology editors, the Academic Editor, and two of the original reviewers.

You'll see that reviewer #1 is now largely satisfied, but has a few residual points, some of which might require minor analyses. Reviewer #2 is also mostly happy, but still requests additional analyses to rule out cross-contamination; it would be good if you could further allay these concerns.

In light of the reviews, which you will find at the end of this email, we are pleased to offer you the opportunity to address the remaining points from the reviewers in a revision that we anticipate should not take you very long. We will then assess your revised manuscript and your response to the reviewers' comments with our Academic Editor aiming to avoid further rounds of peer-review, although we might need to consult with the reviewers, depending on the nature of the revisions.

In addition to these revisions, you may need to complete some formatting changes, which you will receive in a follow up email. A member of our team will be in touch with a set of requests shortly. If you do not receive a separate email within a few days, please assume that checks have been completed, and no additional changes are required.

We expect to receive your revised manuscript within 1 month. Please email us (plosbiology@plos.org) if you have any questions or concerns, or would like to request an extension.

At this stage, your manuscript remains formally under active consideration at our journal; please notify us by email if you do not intend to submit a revision so that we withdraw the manuscript.

**IMPORTANT - SUBMITTING YOUR REVISION**

Your revisions should address the specific points made by each reviewer. Please submit the following files along with your revised manuscript:

1. A 'Response to Reviewers' file - this should detail your responses to the editorial requests, present a point-by-point response to all of the reviewers' comments, and indicate the changes made to the manuscript.

*NOTE: In your point-by-point response to the reviewers, please provide the full context of each review. Do not selectively quote paragraphs or sentences to reply to. The entire set of reviewer comments should be present in full and each specific point should be responded to individually.

You should also cite any additional relevant literature that has been published since the original submission and mention any additional citations in your response.

2. In addition to a clean copy of the manuscript, please also upload a 'track-changes' version of your manuscript that specifies the edits made. This should be uploaded as a "Revised Article with Changes Highlighted " file type.

*Resubmission Checklist*

When you are ready to resubmit your revised manuscript, please refer to this resubmission checklist: https://plos.io/Biology_Checklist

To submit a revised version of your manuscript, please go to https://www.editorialmanager.com/pbiology/ and log in as an Author. Click the link labelled 'Submissions Needing Revision' where you will find your submission record.

Please make sure to read the following important policies and guidelines while preparing your revision:

*Published Peer Review*

Please note while forming your response, if your article is accepted, you may have the opportunity to make the peer review history publicly available. The record will include editor decision letters (with reviews) and your responses to reviewer comments. If eligible, we will contact you to opt in or out. Please see here for more details:

https://blogs.plos.org/plos/2019/05/plos-journals-now-open-for-published-peer-review/

*PLOS Data Policy*

Please note that as a condition of publication PLOS' data policy (http://journals.plos.org/plosbiology/s/data-availability) requires that you make available all data used to draw the conclusions arrived at in your manuscript. If you have not already done so, you must include any data used in your manuscript either in appropriate repositories, within the body of the manuscript, or as supporting information (N.B. this includes any numerical values that were used to generate graphs, histograms etc.). For an example see here: http://www.plosbiology.org/article/info%3Adoi%2F10.1371%2Fjournal.pbio.1001908#s5

*Blot and Gel Data Policy*

We require the original, uncropped and minimally adjusted images supporting all blot and gel results reported in an article's figures or Supporting Information files. We will require these files before a manuscript can be accepted so please prepare them now, if you have not already uploaded them. Please carefully read our guidelines for how to prepare and upload this data: https://journals.plos.org/plosbiology/s/figures#loc-blot-and-gel-reporting-requirements

*Protocols deposition*

To enhance the reproducibility of your results, we recommend that if applicable you deposit your laboratory protocols in protocols.io, where a protocol can be assigned its own identifier (DOI) such that it can be cited independently in the future. Additionally, PLOS ONE offers an option for publishing peer-reviewed Lab Protocol articles, which describe protocols hosted on protocols.io. Read more information on sharing protocols at https://plos.org/protocols?utm_medium=editorial-email&utm_source=authorletters&utm_campaign=protocols

Thank you again for your submission to our journal. We hope that our editorial process has been constructive thus far, and we welcome your feedback at any time. Please don't hesitate to contact us if you have any questions or comments.

Sincerely,

Roli

Roland Roberts, PhD

Senior Editor

PLOS Biology

rroberts@plos.org

----------------------------------------------------------------

REVIEWERS' COMMENTS:

Reviewer #1:

[identifies himself as David Rasmussen]

The authors have addressed my previous concerns and performed additional analyses (e.g. masking sites undergoing convergent evolution) that provide compelling evidence that parallel mutations are driving phylogenetic errors.

Minor comments:

In the Introduction it might be good to cite relevant inference frameworks when introducing the field of phylodynamics.

Throughout: "phylodynamics inference" should probably be "phylodynamic inference".

Discussion line 242: "Thus, the distributions of migration rate estimates in Fig 6 can also be interpreted as the distribution of the inaccuracy of the reconstructed phylogenetic trees." -- I agree that the distribution of migration rates in some way quantifies the inaccuracy of phylogenetic reconstruction. But maybe it would be more clear to link the number of imputed migration rates to the number of discordant edges (i.e. subtree-prune-regraft moves) between the true and reconstructed trees? I think this would be more precise way to quantify inaccuracy since every edge imputed to have a migration event must result from a reconstruction error.

Discussion lines 250-252: "it will be extremely challenging to disentangle whether mutations co-occur in multiple locations due to parallel". -- It may be difficult but probably not impossible. If most polymorphic sites are not undergoing convergent evolution, one could reconstruct a first-pass tree from the full alignment and then use that initial tree to identify sites with a high degree of homoplasy i.e. convergent evolution. One could then filter or mask these sites from the alignment to get an improved phylogenetic reconstruction. Similar approaches are already used to identify and mask recombination events.

Discussion lines 285-287" "however, recombination is unlikely to significantly distort the inferred evolutionary 285 histories because the genotypes in our experiment are highly related, and correct phylogenies can be successfully inferred in such a regime" -- I'm not sure I agree. Phylogenetic inference is generally the most challenging when genetic diversity is low such that recombination becomes difficult to detect and therefore impossible to account for.

Reviewer #2:

First, the authors have done a great job of addressing many of my concerns.

My main remaining concern is about cross-contamination and parallel evolution. I acknowledge the experimental controls described in the manuscript (page 73), and I agree that they make contamination less likely. However, they do not rule it out. The manuscript would be considerably strengthened by explicitly demonstrating that the repeated multi-mutation genotypes represent independent evolutionary events rather than shared ancestry due to contamination. The authors have a simple way to test this using their existing data, so why not do it?

The key point is that cross-contamination and parallel evolution make fundamentally different predictions at the whole-genome level. Cross-contamination transfers complete genomes. If an evolved virus from one evolution line is accidentally introduced into another, it carries with it its entire evolutionary history: not only the focal adaptive mutations, but also every other mutation that accumulated in that lineage, including neutral and hitchhiking variants. In contrast, genuine parallel evolution is expected to produce repeated adaptive mutations on independent genetic backgrounds. Selection may repeatedly favour mutations in the same genes, or even the same combinations of mutations, but the remainder of the genome should differ because each evolution line has accumulated its own independent set of mutations.

Because the authors have whole-genome haplotype information, they are uniquely positioned to distinguish between these alternatives. For each repeated multi-mutation genotype, they should simply compare the complete mutational profiles across the independent evolution lines. If the genomes observed in different evolution lines are genetically indistinguishable across the complete genome, and have multiple differences to the ancestral genome, this would be difficult to reconcile with independent evolution and would instead be more parsimoniously explained by cross-contamination or sample mix-up. Conversely, if the shared adaptive mutations occur within otherwise distinct genomic backgrounds, this would provide compelling direct evidence that the mutations arose independently and would substantially strengthen the claim of parallel evolution.

My concern is that the manuscript makes the unusually strong claim of repeated evolution of whole-genome genotypes, yet the one analysis that would most directly distinguish independent parallel evolution from contamination is not presented. Instead, the manuscript relies on indirect arguments against contamination (experimental controls, spatial separation of evolution lines, genotype frequencies, etc.). These observations do not answer the central genomic question that the authors are uniquely positioned to address with their existing whole-genome data.

This strikes me as a straightforward analysis that can be performed using the existing sequencing data. Given the strength of the claim, I think presenting this analysis explicitly would greatly strengthen the conclusions and remove any lingering ambiguity regarding contamination. At present, this critical distinction is not evident from either the manuscript or the response to reviewers.

Minor points:

In the abstract, why not simply state that you follow the genetic diversification of four bacteriophage populations for 412 generations? You cannot rule out that selection is acting on the population. At present, the authors emphasize that they are not actively imposing selection, while simultaneously acknowledging that selection is probably operating through the bacterial cell environment. Moreover, the principal conclusion of the study is that selection is indeed shaping the evolutionary trajectories, so this repeated qualification seems unnecessary.

I agree with the authors that Figure 4 presents an exceptionally rich and novel dataset, however, in my veiw, I do not think it is appropriate to state that evolution is observed in "unprecedented detail," as this is an unverifiable claim.

Line 202: typo — "ancenstor".

# Author comment: Author response to Decision Letter 4

**DOI:** 10.1371/journal.pbio.3004064.r006

15 Sep 2026

Attachment Submitted filename: responses-to-reviewers-PB-rev2.pdf

# Editor report: Decision Letter 4

**DOI:** 10.1371/journal.pbio.3004064.r007

**Authors:** Roland G Roberts

17 Sep 2026

Dear Roland,

Thank you for your patience while we considered your revised manuscript "Parallel evolution of full-length genomes in a long-term evolution experiment with phage φX174" for publication as a Research Article at PLOS Biology. This revised version of your manuscript has been evaluated by the PLOS Biology editors and the Academic Editor.

Based on our Academic Editor's assessment of your revision, we are likely to accept this manuscript for publication, provided you satisfactorily address the following editorial, data and other policy-related requests.

IMPORTANT - please attend to the following:

a) We need your Title to contain an active verb; I suggest flipping it around to something like this: "A long-term experiment with phage φX174 reveals parallel evolution of full-length genomes" (I've removed a second instance of the word "evolution" to avoid repetition, but I can understand that you might want to retain it, perhaps to chime with the Lenski LTEE)

b) I spotted a couple of errors; on line 203, you should presumably cite new Fig S4 (it's blank in my version), and the legend for Fig 5 should read “and combinations of mutations (B–F),” not “B-G.”

c) Please address my Data Policy requests below; specifically, we need you to supply the numerical values underlying Figs 2ABC, 3BCD, 4A, 5ABCDEF, 6ABCD, S1, S2AB, S3, S4, either as a supplementary data file or as a permanent DOI’d deposition. I note that you already have an associated Zenodo deposition (https://doi.org/10.5281/zenodo.22711242). Please could you confirm whether the data and code in this deposition are sufficient to recreate the Figures? Please also make it accessible so that I can check that it is compliant with our data policy.

d) Please cite the location of the data clearly in all relevant main and supplementary Figure legends, e.g. “The data and code needed to reproduce this Figure can be found in https://zenodo.org/records/22711242"

e) Please make any custom code available, either as a supplementary file or as part of your data deposition.

f) Please include the URLs of your funders in the Financial Disclosure statement.

As you address these items, please take this last chance to review your reference list to ensure that it is complete and correct. If you have cited papers that have been retracted, please include the rationale for doing so in the manuscript text, or remove these references and replace them with relevant current references. Any changes to the reference list should be mentioned in the cover letter that accompanies your revised manuscript.

In addition to these revisions, you may need to complete some formatting changes, which you will receive in a follow up email. A member of our team will be in touch with a set of requests shortly. If you do not receive a separate email within a few days, please assume that checks have been completed, and no additional changes are required.

We expect to receive your revised manuscript within two weeks.

To submit your revision, please go to https://www.editorialmanager.com/pbiology/ and log in as an Author. Click the link labelled 'Submissions Needing Revision' to find your submission record. Your revised submission must include the following:

- a cover letter that should detail your responses to any editorial requests, if applicable, and whether changes have been made to the reference list

- a Response to Reviewers file that provides a detailed response to the reviewers' comments (if applicable, if not applicable please do not delete your existing 'Response to Reviewers' file.)

- a track-changes file indicating any changes that you have made to the manuscript.

NOTE: If Supporting Information files are included with your article, note that these are not copyedited and will be published as they are submitted. Please ensure that these files are legible and of high quality (at least 300 dpi) in an easily accessible file format. For this reason, please be aware that any references listed in an SI file will not be indexed. For more information, see our Supporting Information guidelines:

https://journals.plos.org/plosbiology/s/supporting-information

*Published Peer Review History*

Please note that you may have the opportunity to make the peer review history publicly available. The record will include editor decision letters (with reviews) and your responses to reviewer comments. If eligible, we will contact you to opt in or out. Please see here for more details:

https://plos.org/published-peer-review-history/

*Press*

Should you, your institution's press office or the journal office choose to press release your paper, please ensure you have opted out of Early Article Posting on the submission form. We ask that you notify us as soon as possible if you or your institution is planning to press release the article.

*Protocols deposition*

To enhance the reproducibility of your results, we recommend that if applicable you deposit your laboratory protocols in protocols.io, where a protocol can be assigned its own identifier (DOI) such that it can be cited independently in the future. Additionally, PLOS ONE offers an option for publishing peer-reviewed Lab Protocol articles, which describe protocols hosted on protocols.io. Read more information on sharing protocols at https://plos.org/protocols?utm_medium=editorial-email&utm_source=authorletters&utm_campaign=protocols

Please do not hesitate to contact me should you have any questions.

Sincerely,

Roli

Roland Roberts, PhD

Senior Editor

rroberts@plos.org

PLOS Biology

------------------------------------------------------------------------

DATA POLICY:

You may be aware of the PLOS Data Policy, which requires that all data be made available without restriction: http://journals.plos.org/plosbiology/s/data-availability. For more information, please also see this editorial: http://dx.doi.org/10.1371/journal.pbio.1001797

Note that we do not require all raw data. Rather, we ask that all individual quantitative observations that underlie the data summarized in the figures and results of your paper be made available in one of the following forms:

1) Supplementary files (e.g., excel). Please ensure that all data files are uploaded as 'Supporting Information' and are invariably referred to (in the manuscript, figure legends, and the Description field when uploading your files) using the following format verbatim: S1 Data, S2 Data, etc. Multiple panels of a single or even several figures can be included as multiple sheets in one excel file that is saved using exactly the following convention: S1_Data.xlsx (using an underscore).

2) Deposition in a publicly available repository. Please also provide the accession code or a reviewer link so that we may view your data before publication.

Regardless of the method selected, please ensure that you provide the individual numerical values that underlie the summary data displayed in the following figure panels as they are essential for readers to assess your analysis and to reproduce it: Figs 2ABC, 3BCD, 4A, 5ABCDEF, 6ABCD, S1, S2AB, S3, S4. NOTE: the numerical data provided should include all replicates AND the way in which the plotted mean and errors were derived (it should not present only the mean/average values).

IMPORTANT: Please also ensure that figure legends in your manuscript include information on where the underlying data can be found, and ensure your supplemental data file/s has a legend.

Please ensure that your Data Statement in the submission system accurately describes where your data can be found.

------------------------------------------------------------------------

CODE POLICY

Per journal policy, if you have generated any custom code during the course of this investigation, please make it available without restrictions. Please ensure that the code is sufficiently well documented and reusable, and that your Data Statement in the Editorial Manager submission system accurately describes where your code can be found. More information on our Code Policy, what and how to share can be found here: https://journals.plos.org/plosbiology/s/code-availability

Please note that we cannot accept sole deposition of code in GitHub, as this could be changed after publication. However, you can archive this version of your publicly available GitHub code to Zenodo. Once you do this, it will generate a DOI number, which you will need to provide in the Data Accessibility Statement (you are welcome to also provide the GitHub access information). See the process for doing this here: https://docs.github.com/en/repositories/archiving-a-github-repository/referencing-and-citing-content

------------------------------------------------------------------------

DATA NOT SHOWN?

- Please note that per journal policy, we do not allow the mention of "data not shown", "personal communication", "manuscript in preparation" or other references to data that is not publicly available or contained within this manuscript. Please either remove mention of these data or provide figures presenting the results and the data underlying the figure(s).

------------------------------------------------------------------------

# Author comment: Author response to Decision Letter 5

**DOI:** 10.1371/journal.pbio.3004064.r008

7 Oct 2026

Attachment Submitted filename: responses-to-reviewers-PB-rev2_auresp_5.pdf

# Editor report: Decision Letter 5

**DOI:** 10.1371/journal.pbio.3004064.r009

**Authors:** Roland G Roberts

8 Oct 2026

Dear Roland,

Thank you for the submission of your revised Research Article "Full-length genomes evolve in parallel in a long-term evolution experiment with phage φX174" for publication in PLOS Biology. On behalf of my colleagues and the Academic Editor, Jeremy Barr, I'm pleased to say that we can in principle accept your manuscript for publication, provided you address any remaining formatting and reporting issues. These will be detailed in an email you should receive within 2-3 business days from our colleagues in the journal operations team; no action is required from you until then. Please note that we will not be able to formally accept your manuscript and schedule it for publication until you have completed any requested changes.

Please take a minute to log into Editorial Manager at http://www.editorialmanager.com/pbiology/, click the "Update My Information" link at the top of the page, and update your user information to ensure an efficient production process.

PRESS: We frequently collaborate with press offices. If your institution or institutions have a press office, please notify them about your upcoming paper at this point, to enable them to help maximise its impact. If the press office is planning to promote your findings, we would be grateful if they could coordinate with biologypress@plos.org. If you have previously opted in to the early version process, we ask that you notify us immediately of any press plans so that we may opt out on your behalf.

We also ask that you take this opportunity to read our Embargo Policy regarding the discussion, promotion and media coverage of work that is yet to be published by PLOS. As your manuscript is not yet published, it is bound by the conditions of our Embargo Policy. Please be aware that this policy is in place both to ensure that any press coverage of your article is fully substantiated and to provide a direct link between such coverage and the published work. For full details of our Embargo Policy, please visit http://www.plos.org/about/media-inquiries/embargo-policy/.

Thank you again for choosing PLOS Biology for publication and supporting Open Access publishing. We look forward to publishing your study.

Sincerely,

Roli

Roland G Roberts, PhD, PhD

Senior Editor

PLOS Biology

rroberts@plos.org
